# Electrode-associated growth reveals structure of OmcZ-type cytochrome nanowires in anaerobic methanotrophic archaea

**DOI:** 10.1038/s41467-026-78976-x

**Published:** 2026-11-09

**Authors:** Martijn Wissink, Cong Shen, Jiaqi Wang, Reinier A. Egas, Phoenix Smid, Felix J. Elling, Kevin W. Becker, Martin Pabst, Mike S. M. Jetten, Jun Liu, Nikhil S. Malvankar, Cornelia U. Welte

**Affiliations:** 1 Department of Microbiology, Radboud Institute for Biological and Environmental Sciences, Radboud University, Nijmegen, The Netherlands; 2 Microbial Sciences Institute, Yale University, West Haven, CT USA; 3 Department of Molecular Biophysics & Biochemistry, Yale University, New Haven, CT USA; 4 Department of Microbial Pathogenesis, Yale University, New Haven, CT USA; 5 Leibniz-Laboratory for Radiometric Dating and Isotope Research, Christian-Albrechts-University of Kiel, Kiel, Germany; 6 Faculty of Biology/Chemistry, University of Bremen, Bremen, Germany; 7 Department of Biotechnology, Delft University of Technology, Delft, The Netherlands

**Keywords:** Archaeal physiology, Biophysics, Cryoelectron microscopy, Microbial ecology

## Abstract

Anaerobic methanotrophic (ANME) archaea are key regulators of methane emissions in anoxic environments, yet their physiology remains poorly understood due to the lack of axenic cultures. Here, we show that cultivation on poised electrodes yields near-axenic cultures (95.8% relative abundance) of a freshwater ANME, for which we propose the name ‘*Candidatus* Methanoperedens vercellensis’. We identified key physiological properties of ‘*Ca*. M. vercellensis’: doubling times of 4.6 days at 30 °C, menaquinone as respiratory electron carrier, the inability to oxidize ethane, and preferential reduction of iron oxides over nitrate after cultivation on electrodes. A 3.3 Å cryo-electron microscopy structure of filaments displayed on the archaeal surface revealed polymerized OmcZ-type cytochrome nanowires, which are homologous to OmcZ nanowires essential for long-range (>10 μm) extracellular electron transfer (EET) in *Geobacter sulfurreducens*. The archaeal OmcZ-type nanowires include dense, collinear stacking of protected hemesakin to bacterial OmcZ nanowires, but with post-translational modification, thus yielding morphology distinct from bacterial OmcZ nanowires. Notably, in contrast to bacterial OmcZ, archaeal OmcZ-type nanowires do not form interconnected networks, and hemes are less solvent-exposed, which can protect them from the environment. Surprisingly, these archaea assemble OmcZ into nanowires even in the apparent absence of prolyl isomerase involved in folding bacterial OmcZ. This work establishes a model system for molecular understanding of nanowire-mediated EET in archaea.

## Abstract (web summary)

Anaerobic methanotrophic archaea are key regulators of methane emissions in anoxic environments. Here, the authors report near-axenic cultivation of these microorganisms on electrodes, describing key physiological traits and presence of OmcZ-type cytochrome nanowires likely involved in extracellular electron transfer.

## Introduction

Methane is a potent greenhouse gas with a global warming potential approximately 28 times greater than that of carbon dioxide over a 100-year timescale, making its biological oxidation an important natural process for climate regulation^1–3^. Anaerobic methane-oxidizing (ANME) archaea consume a substantial fraction (3–71%) of methane emissions and play a central role in the cycling of elements such as nitrogen and iron^4,5^. In marine ecosystems, ANME form syntrophic partnerships with sulfate-reducing bacteria (SRB) to couple methane oxidation to sulfate reduction^6,7^ whereas in freshwater ecosystems, ANME of the genus “*Candidatus* Methanoperedens” couple methane oxidation to the reduction of alternative electron acceptors, including nitrate, and iron and manganese oxides^8–11^.

Despite their global importance, in-depth physiological studies of ANME remain scarce. This is largely due to the absence of axenic cultures, which necessitates studying ANME within complex microbial communities, combined with inherently slow growth rates ranging from 19 days to 7.5 months^12–15^. Although next-generation sequencing has greatly expanded our understanding of ANME metabolic potential, genomic data alone provide an incomplete picture of function^16–18^. For example, ANME genomes encode five- to eight-fold more multiheme c-type cytochromes (MHCs) than their methanogenic relatives^16^, yet the physiological roles of these proteins remain poorly understood. In marine systems, high rates of anaerobic methane oxidation are proposed to be enabled by direct interspecies electron transfer (DIET) mediated by MHCs and conductive “nanowires” between ANME and SRB^6,7^. However, the exact molecular components and pathways underlying extracellular electron transfer (EET) and methane oxidation in ANME remain largely unresolved.

To overcome these limitations and directly probe the mechanisms underlying EET in ANME, bioelectrochemical systems (BESs) have emerged as a powerful experimental platform^19^. By providing a poised electrode as an alternative electron acceptor, a selective environment is created that enables a controlled investigation of metabolic processes. Applying this approach to freshwater ANME-2d “*Ca*. Methanoperedens” has demonstrated methane-dependent current production and implicated MHCs as key components for EET^20–22^.

Functional nanowires involved in EET have not yet been demonstrated in archaea. A recent study reported cryo-electron microscopy (cryo-EM) structures of cytochrome filaments from *Pyrobaculum calidifontis* (ECN^Pc^) and *Archaeoglobus veneficus* (ECN^Av^)^23^. However, the physiological roles of these filaments remain unresolved, as these archaea were neither grown on electrodes nor with extracellular electron acceptors, and no intact cell-attached filaments have been reported.

In contrast, our current understanding of the role of MHCs in microbial EET mechanisms originates predominantly from studies of electroactive bacteria, particularly *Shewanella* and *Geobacter* species. *Shewanella* species use the Mtr respiratory pathway, which includes outer membrane cytochrome proteins for the reduction of extracellular electron acceptors, including electrodes^24^. In *Geobacter* species, conductive surface-displayed nanowires play a key role in EET. Although their molecular identity was long debated, accumulating evidence now supports polymerized MHCs of OmcS^25,26^ and OmcZ^27,28^ as the primary components of nanowires for EET. Additionally, in *omcS*-deficient mutant strains, OmcE can polymerize into nanowires in vitro^29^, although the physiological relevance of these structures remains unclear.

Out of these MHCs, OmcZ is the only outer-surface nanowire-forming cytochrome known to be essential for long-range (>10 µm) EET in microbial communities grown on electrodes^27,30^. Among reported cytochrome nanowires, OmcZ uniquely features linear, compact heme arrangements that confer high conductivity^27,30^. Notably, OmcZ homologs seem more widespread in archaea than in bacteria and are frequently found in genomes of “*Ca*. Methanoperedens” and in giant (>1 Mb) extrachromosomal elements called Borgs that are associated with “*Ca*. Methanoperedens” genomes^31,32^. Moreover, genomic neighborhoods of OmcZ homologs often encode OzpA (OmcZ protease) homologs for nanowire maturation^33^. Consistent with these genomic findings, OmcZ homologs are actively expressed in “*Ca*. Methanoperedens” during metal-dependent respiration: a 44% identity OmcZ-type homolog was highly expressed during Fe(III) reduction in “*Ca*. Methanoperedens nitroreducens”^22^, and a 21% identity homolog was among the most abundant transcripts during Fe(III) reduction in “*Ca*. Methanoperedens ferrireducens”^8^. Similarly, OmcZ homologs were among the top-expressed genes when “*Ca*. Methanoperedens” was found in electrode-associated microbial communities^21^. Despite these transcriptomic-level observations, it remains unclear whether these archaeal OmcZ homologs assemble into functional nanowires or how they contribute to EET.

In this study, we employed BESs to cultivate “*Ca*. Methanoperedens” under controlled electrochemical conditions and obtained a near-axenic culture (95.8% relative abundance) of “*Candidatus* Methanoperedens sp.” Vercelli strain 1, for which we propose the name “*Candidatus* Methanoperedens vercellensis”. This strain harbors a large plasmid, but no Borg-like extrachromosomal elements^34^. Using this near-axenic culture, we determined key physiological parameters and found that EET-performing archaeal cells produce OmcZ-type cytochrome nanowires. Cryo-EM reveals structural features that we hypothesize may be optimized for high electron conductivity and efficient EET, distinct from *Geobacter sulfurreducens* OmcZ.

## Results

### Bioelectrochemical cultivation yields a near-axenic anaerobic methanotroph culture

**Fig. 1:**
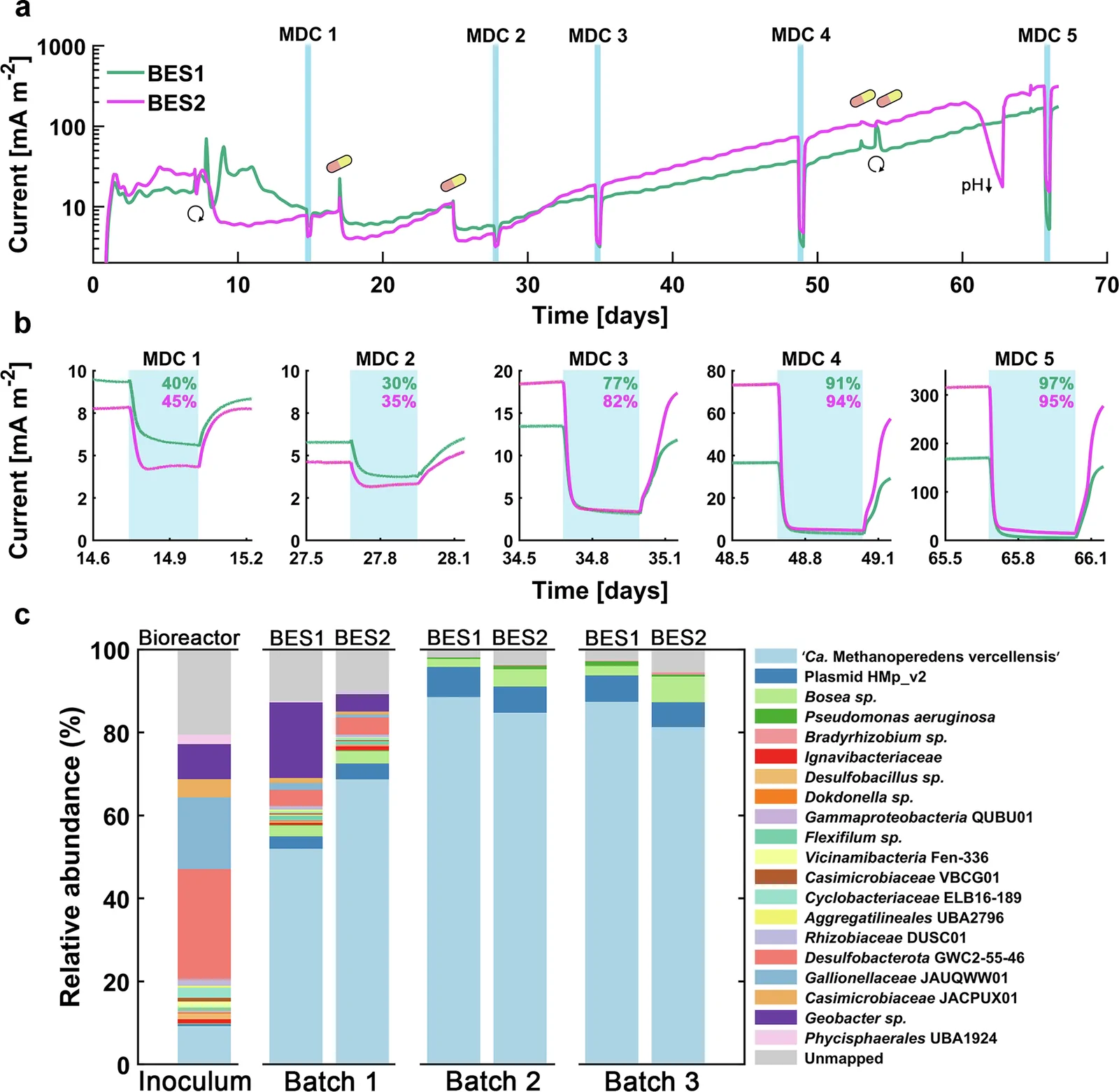
Methane-dependent current generation and microbial community composition of bioelectrochemical systems. **a** Current generation over time in duplicate BES experiments. Medium replacements are indicated by circular arrows, and the addition of bacteria-suppressing antibiotics is shown by purple-yellow symbols. Periods in which the methane supply was replaced with argon to test for methane-dependent current are marked with blue bars. The drop in current at day 60 in BES2 was caused by a decrease in pH, due to high methanotrophic activity. Amending the pH restored the current. After 67 days the duplicate BESs were subcultured into new BESs. **b** Zoom-in of the methane-dependent current test periods shown in (**a**). The percentages shown in green (BES1) and pink (BES2) represent the proportion of current attributed to methane oxidation. **c** Microbial community composition of the bioreactor inoculum and duplicate BESs at the end of three consecutive batch cultivations. Short-read sequence reads were mapped to metagenome-assembled genomes (MAGs) to determine relative abundances. Only MAGs with >40% completeness and <10% contamination were included in the analysis. For full taxonomic classification see Supplementary Data 1. Source data are provided as a Source Data file.

“*Ca*. Methanoperedens” was cultivated in duplicate BESs with methane as the sole electron donor and a carbon felt electrode as the sole electron acceptor for 352 consecutive days. Every ~70 days, the electrodes were subcultured into new BESs, showing continued exponential current production, signifying microbial growth (Fig. 1a and Supplementary Fig. 1). The methane dependency of the produced current was very high, reaching 97% (Fig. 1b), indicating that essentially all current was produced through methane oxidation. The remaining current in the absence of methane could originate from switching to oxidation of intracellular storage compounds such as polyhydroxyalkanoates (PHAs) by “*Ca*. Methanoperedens”^35^. At 280 days, the coulombic efficiency was 93 ± 8% (mol electrons generated/mol methane oxidized) (Supplementary Fig. 2A), far exceeding the previously reported 17%^21^, indicating that all the consumed methane is essentially converted into current, with only a small percentage of electrons used for anabolic processes.

To assess the microbial community structure during cultivation, we performed metagenomic sequencing of the bioreactor inoculum and BES cultivations at days 67, 119, and 207. Combining short- and long-read sequencing enabled us to obtain a complete genome sequence of “*Ca*. Methanoperedens sp.” Vercelli Strain 1 (Supplementary Table 1), consisting of a circular chromosome of 3.41 Mbp. Together with the circular chromosome, the 156 Kbp plasmid HMp_v2 was found at a 1:1 ratio to the genome, in line with previous investigations of extrachromosomal elements associated with this strain^34^. Closing the genome revealed an additional 60 protein-coding sequences, including five predicted multiheme *c*-type cytochromes (MHC; ≥3 CxxCH, C3xCH, or CxxCK motifs). Having closed its genome, we now propose the name “*Ca*. M. vercellensis” for this strain.

The bioreactor inoculum contained 9.7% “*Ca*. M. vercellensis” based on read mapping to metagenome-assembled genomes (MAG) (Fig. 1c). The two biological replicates had a relative abundance of “*Ca*. M. vercellensis” including plasmid HMp_v2 of 55% and 73% after 67 days, of 96% and 91% after 119 days, and of 94% and 87% after 207 days, indicating a stabilization of community composition and establishment of a growing, near-axenic “*Ca*. M. vercellensis” culture after 119 days. At the same time, the well-known electrogen *Geobacter* sp. was present at 18% and 4.2% relative abundance in both replicates after 67 days, compared to 8.5% in the bioreactor inoculum. However, after 119 days, *Geobacter* sp. were absent from the microbial community, which was confirmed by negative-stain TEM images (Supplementary Fig. 3). *Geobacter* sp. likely established a biofilm in the BES before the addition of the bacteria-suppressing antibiotics, and necromass DNA accounted for its initial detection in the metagenomics dataset.

Apart from “*Ca*. M. vercellensis”, another microbe, a *Bosea* sp. bacterium, increased substantially in relative abundance during cultivation in BESs to reach the highest observed relative abundance of 6.2% in BES2 after 207 days (Fig. 1c). The Gram-negative genus *Bosea* is strictly aerobic and can oxidize various carbon sources and amino acids^36–38^. The *Bosea* MAG obtained from the electrodes did not encode any characterized EET pathways (OmcS, OmcZ, OmcE, Mtr) or methane activation machinery (MCR, pMMO, sMMO). It likely thrives on trace oxygen amounts leaking into the BES, as well as carbon exudates or necromass from “*Ca*. M. vercellensis”. Therefore, it is unlikely that *Bosea* sp. directly participates in methane oxidation on the electrode; it might, however, provide unknown essential metabolites to “*Ca*. M. vercellensis”.

### Key physiological properties of electrode-grown “*Ca*. M. vercellensis”

**Fig. 2:**
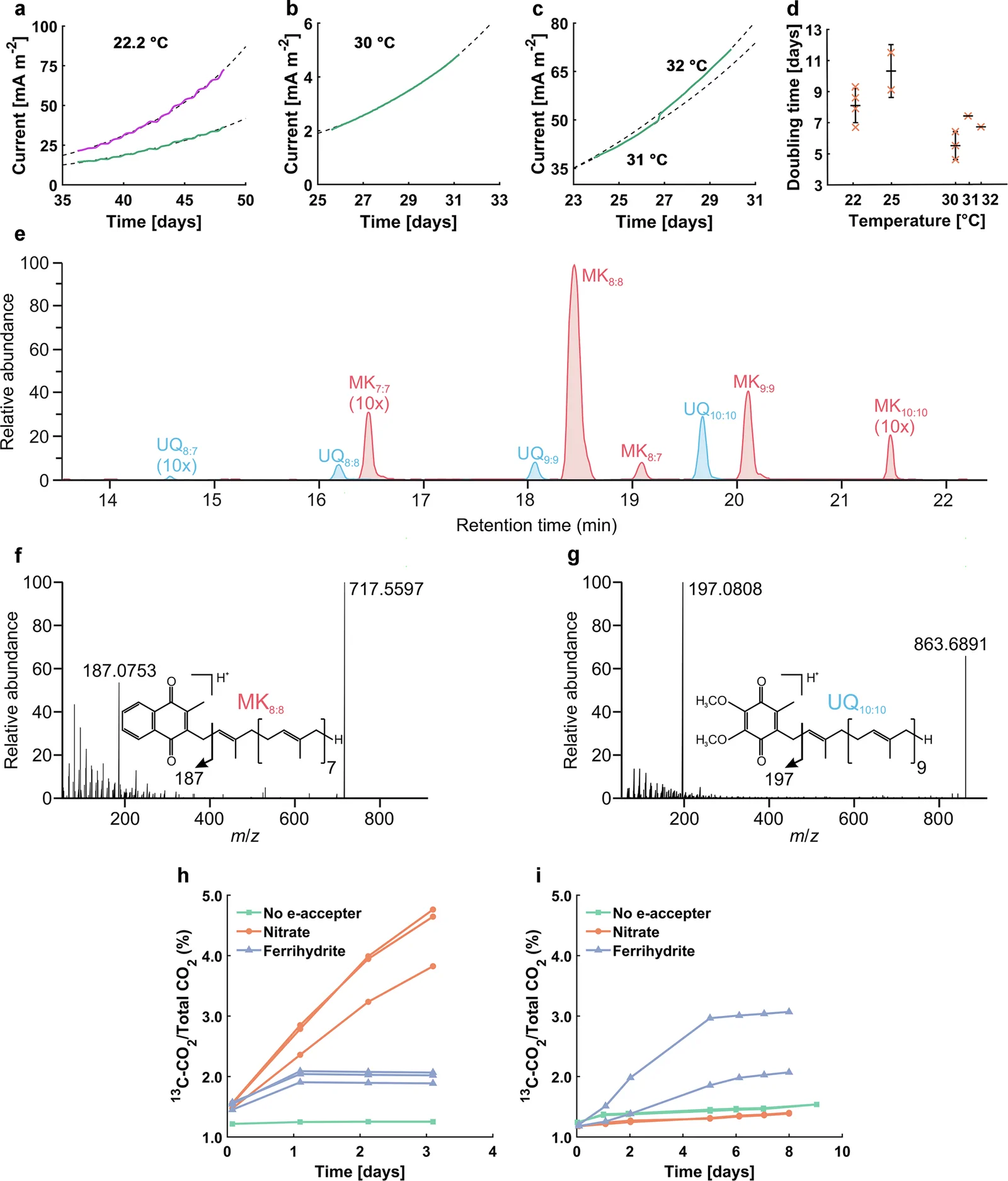
Physiological properties of electrode-grown “*Ca*. M. vercellensis”. **a**–**d** Exponential current increase as a proxy for growth. **a**–**c** Current production at different temperatures (biological duplicates in pink and green or Supplementary Fig. 2) with fitted exponential trendline (black dashed line). **c** On day 27 the temperature was increased from 31 °C to 32 °C. **d** Current doubling time as a function of incubation temperature. Individual measurements (orange ×) are shown together with the mean ± SD (black bars) (Biological replicates for 22 °C *n* = 4; 25 °C *n* = 2; 30 °C *n* = 3, 31 °C *n* = 1, 32 °C *n* = 1). **e**–**g** Quinone composition of the electrode-associated community. **e** Extracted ion chromatogram of ubiquinones (UQ) and menaquinones (MK) with varying side-chain lengths and degrees of unsaturation. Low-abundance peaks were vertically exaggerated 10x. **f** MS² spectrum of MK_8:8_ ([M + H]⁺ = 715.5449; diagnostic fragment = 187.0754). **g** MS² spectrum of UQ_10:10_ ([M + H]⁺ = 863.6912; diagnostic fragment = 197.0808). Quinone nomenclature (Qₘ_:_ₙ) denotes quinone type (Q), number of isoprenoid units (m), and double bonds (n). Additional quinones are listed in Supplementary Table 2. **h**, **i** ^13^C-CH_4_ oxidation rates before and after electrode incubation. **h** Bioreactor inoculum incubated without electron acceptor (green), with nitrate (orange), or ferrihydrite (blue) (in biological triplicate, *n* = 3; all measured data points are shown, individual experimental treatments are connected by a solid line). **i** Electrode-associated community after 289 days of BES incubation under the same conditions (in biological duplicate, *n* = 2; error bars indicate standard deviation). Source data are provided as a Source Data file.

Fitting an exponential growth curve to the data of the first 67 days yielded a current doubling time of 8.1 ± 1.1 days (Fig. 2a). Given that >95% of the current is derived from methane oxidation with a coulombic efficiency of 93 ± 8%, and that >90% of the microbial community consists of “*Ca*. M. vercellensis”, the doubling time of the current can be interpreted as a proxy for the doubling time of “*Ca*. M. vercellensis”. Following the initial BES incubations at room temperature (22.2 °C), we investigated the effect of elevated temperatures on current production. Implementing temperature control not only stabilized the signal by reducing current fluctuations caused by diurnal temperature changes (Supplementary Fig. 2B, C) but also revealed a temperature-dependent response: increasing the incubation temperature to 30 °C reduced the current doubling time to 5.5 ± 0.9 days (Fig. 2a–c, Supplementary Fig. 2D–G). When plotting doubling time as a function of temperature, a general trend of faster growth at higher temperatures was observed, with an optimum at 30 °C (Fig. 2d).

Methanogens of the Methanosarcinales order typically employ methanophenazine as a redox shuttle in their electron transport chains^39^. Although Methanoperedenaceae belong to the same order, previous analyses have not detected methanophenazine in enrichment cultures^40^, and the corresponding biosynthesis pathway remains unresolved, leaving its presence in the genome of “*Ca*. M. vercellensis” uncertain. Instead, the futalosine pathway for menaquinone synthesis is encoded in the genome (Supplementary Data 1). Consistent with this genomic prediction, lipidomic analysis on the electrode-associated community revealed no detectable methanophenazine, whereas menaquinone was identified as the dominant membrane-integral redox carrier (Fig. 2e, f, Supplementary Table 2), indicating that it functions as the principal electron shuttle in “*Ca*. M. vercellensis”. A minor fraction of the detected quinones consisted of ubiquinones (Fig. 2e, g), which likely originate from *Bosea* sp., the second most abundant organism in the community.

After methane, ethane is the second most abundant hydrocarbon in natural gas escaping from cold seeps and hydrothermal vents^41^. Previous studies have hypothesized that methanotrophic MCRs are incapable of oxidizing ethane^42^, which was tested by replacing methane with ethane in a BES. Ethane addition did not lead to an increase in current beyond the baseline established after argon flushing, indicating that “*Ca*. M. vercellensis” cannot metabolize ethane as an electron donor (Supplementary Fig. 2H, I). After resuming regular flushing with methane, the level of current re-established as before the experiment, indicating that the addition of ethane had not irreversibly inhibited the metabolism of “*Ca*. M. vercellensis”.

To investigate how electrode respiration changes electron acceptor preference in “*Ca*. M. vercellensis”, we compared methane oxidation rates with different electron acceptors between the original bioreactor enrichment culture and the electrode-associated community after 289 days of electrode-associated growth. The original bioreactor culture exhibited higher methane oxidation rates with nitrate than with ferrihydrite (an iron mineral) (Fig. 2h). In contrast, after growth on electrodes, the “*Ca*. M. vercellensis” culture preferred ferrihydrite over nitrate as an electron acceptor, suggesting a preference for EET to insoluble electron acceptors (Fig. 2i). Notably, the electrode-grown culture did not resume methane oxidation in the presence of nitrate during the 8-day incubation period. In the absence of genomic changes, this may indicate that switching back to nitrate respiration is a slow regulatory process, or that nitrite accumulation, resulting from the loss of nitrite-scavenging partners (Supplementary Data 1; genomes encoding dissimilatory nitrite reductases in the inoculum but absent in the BESs were *Gallionellaceae*, *Geobacter*, *Casimicrobiaceae*, *Phycisphaerales*), inhibits nitrate-dependent methane oxidation in the electrode-adapted community.

### The discovery and structure of OmcZ-type nanowires from electrode-grown “*Ca*. M. vercellensis”

**Fig. 3:**
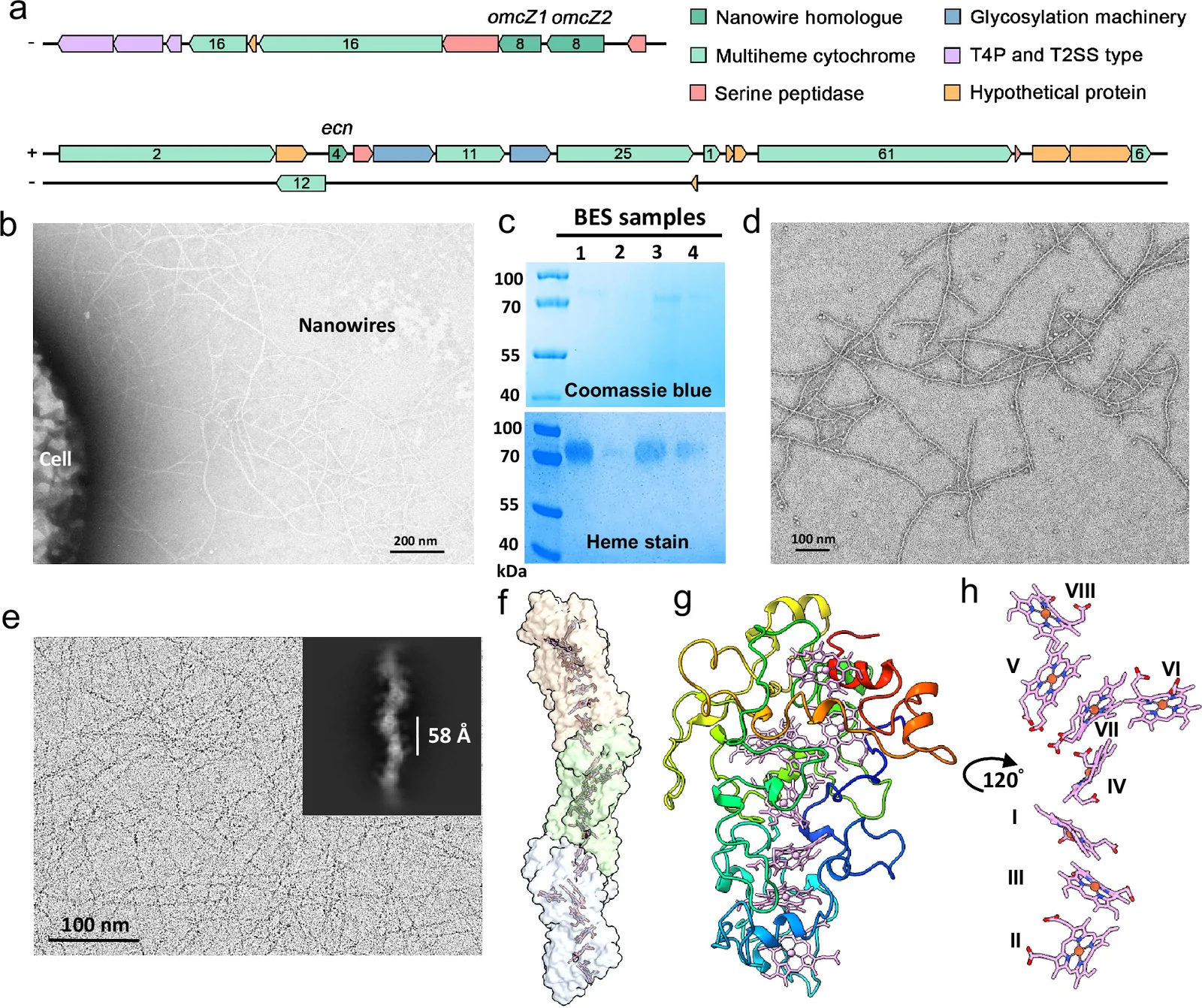
The discovery, purification and atomic structure of the OmcZ2^Mv^ nanowires produced by electrode-grown “*Ca*. M. vercellensis”. **a** Genomic cluster organizations of OmcZ and ECN homologs in “*Ca*. M. vercellensis”. Numbers indicate the number of heme-binding motifs in each gene. T4P refers to archaellin/type IV pilin proteins, and T2SS to type II secretion system proteins. **b** Negative stain TEM image of surface-displayed cytochrome filaments produced by electrode-grown cells; representative micrographs from three rounds of image collection are shown. **c** Coomassie blue (top) and heme (bottom) stained gel images of OmcZ2^Mv^ nanowires in four purification fractions: (1) the electrode-grown culture medium, HEPES based buffer, pH 7.25; (2) the supernatant of 1) after 21,300 × *g* spin; (3) the pellet of 1) after 21,300 × *g* spin and resuspended in 50 mM Tris 50 mM NaCl pH 8.0 buffer; (4) the 10-fold concentrated fraction (2). Due to the limited amount of biomass collected from the electrodes, this experiment had one biological replicate. **d** The negative stain TEM image of purified OmcZ2^Mv^ nanowires. Representative micrographs from three rounds of image collection are shown. **e** Cryo-EM images of purified OmcZ2^Mv^ nanowires. Inset, the two-dimensional class average of OmcZ2^Mv^ nanowires with the 58 Å helical rise. **f** The atomic structure of OmcZ2^Mv^ nanowire revealed a nearly collinear heme chain at its center. **g** The circuitous backbone with blue for the N-terminal part, red for the C-terminal part, and a successive gradient change between them. **h** Hemes numbered as per the protein sequence with nitrogen (blue), oxygen (red), and iron (orange) atoms.

The genome of “*Ca*. M. vercellensis” encodes 39 MHCs, several of which are organized in genomic clusters. Two such clusters contain homologs of known cytochrome nanowires (Fig. 3a). One cluster encodes two homologs of *Geobacter sulfurreducens* OmcZ (OmcZ^Gs^), sharing 34% amino acid identity (99% coverage, *E*-value 7 × 10^−73^) and 29% amino acid identity (74% coverage, *E*-value 2 × 10^−39^), respectively. These proteins were designated OmcZ1^Mv^ (gene ID: DOEMACJB_03675) and OmcZ2^Mv^ (DOEMACJB_03676) and are flanked by two genes encoding serine proteases (Fig. 3a), consistent with the presence of OmcZ-specific proteases that are essential for OmcZ cleavage and nanowire maturation^27,33^. Further downstream, the genomic neighborhood encodes Type II/IV secretion system proteins and Type IV pili subunits (DOEMACJB_03667-03670) which may be involved in secretion of OmcZ as well as anchoring to the cytoplasmic membrane. A second cluster encodes a 4-heme cytochrome (DOEMACJB_01452) with 29% identity (86% coverage, *E*-value 3 × 10^−12^) to the archaeal extracellular cytochrome nanowire (ECN) of *A. veneficus* and was therefore designated ECN^Mv^.

Negative-stain TEM images revealed abundant filaments extending from the cell surfaces of the electrode-grown samples (Fig. 3b; Supplementary Fig. 3). When purified, both Coomassie blue (Fig. 3c) and heme-stain gels (Fig. 3c) showed a single major band at approximately 70 kDa. These filaments exhibited the characteristic sinusoidal feature of cytochrome nanowires (Fig. 3d)^25–27^. Mass spectrometry data revealed OmcZ2^Mv^ as the most abundant cytochrome in both the gel bands and the electrode biofilm (Supplementary Fig. 4A, B; Supplementary Data 2). Other c-type cytochromes were present at very low abundances (Supplementary Data 2). In addition to high protein abundance in the near-axenic “*Ca*. M. vercellensis” culture, OmcZ2^Mv^ was one of the most highly expressed genes in RNA-seqdata from electrode-associated “*Ca*. M. vercellensis” in a previous study, although it appeared truncated without heme-binding motifs in a fragmented genome^21^.

Another type of filament was observed at low abundance that appeared thicker in negative-stain TEM images (Supplementary Fig. 4C, D). However, this filament was not detected in cryo-EM images. While OmcZ1^Mv^ was barely detected in the mass spectrometry data, the presence of this thicker filament coincided with ECN^Mv^ being the second most abundant cytochrome in the electrode medium at a 1:20 ratio compared to OmcZ2^Mv^ (Supplementary Data 2). Although the identity of this filament remains unresolved, its absence from cryo-EM images suggests that OmcZ2^Mv^ is the dominant nanowire involved in electrode-associated EET in “*Ca*. M. vercellensis”.

The dense networks of filaments in cryo-EM images (Fig. 3e) enabled the reconstruction of an atomic model of OmcZ2^Mv^ nanowires. The structure was resolved at 3.3 Å (Supplementary Figs. 5, 6) and revealed a helical assembly with a rise of 58 Å and a twist of 159° per protomer (Fig. 3f). Similar to OmcZ^Gs^, OmcZ2^Mv^ undergoes proteolytic processing in which the C-terminal domain (~30 kDa) is cleaved off, yielding a nanowire-forming N-terminal part of 330 residues containing eight canonical *c*-type hemes (Fig. 3g, h; Supplementary Table 3). In contrast, the nanowire-forming part of OmcZ^Gs^ comprises 258 residues and includes one heme bound by a non-canonical motif (CX_14_CH). In both homologs, about 80% of the structure is composed of turns and loops (Fig. 3g; Supplementary Table 3).

Previous structural studies of OmcZ^Gs^ required alkaline conditions (ethanolamine buffer, pH 10.5) to prevent extensive filament aggregation in vitro^27,28^. In contrast, here the native structure of OmcZ2^Mv^ nanowires was directly resolved under physiological conditions (HEPES-buffered growth medium, pH 7.25), without precipitation by ammonium sulfate.

### Structural features possibly related to high conductivity in OmcZ2^Mv^

**Fig. 4:**
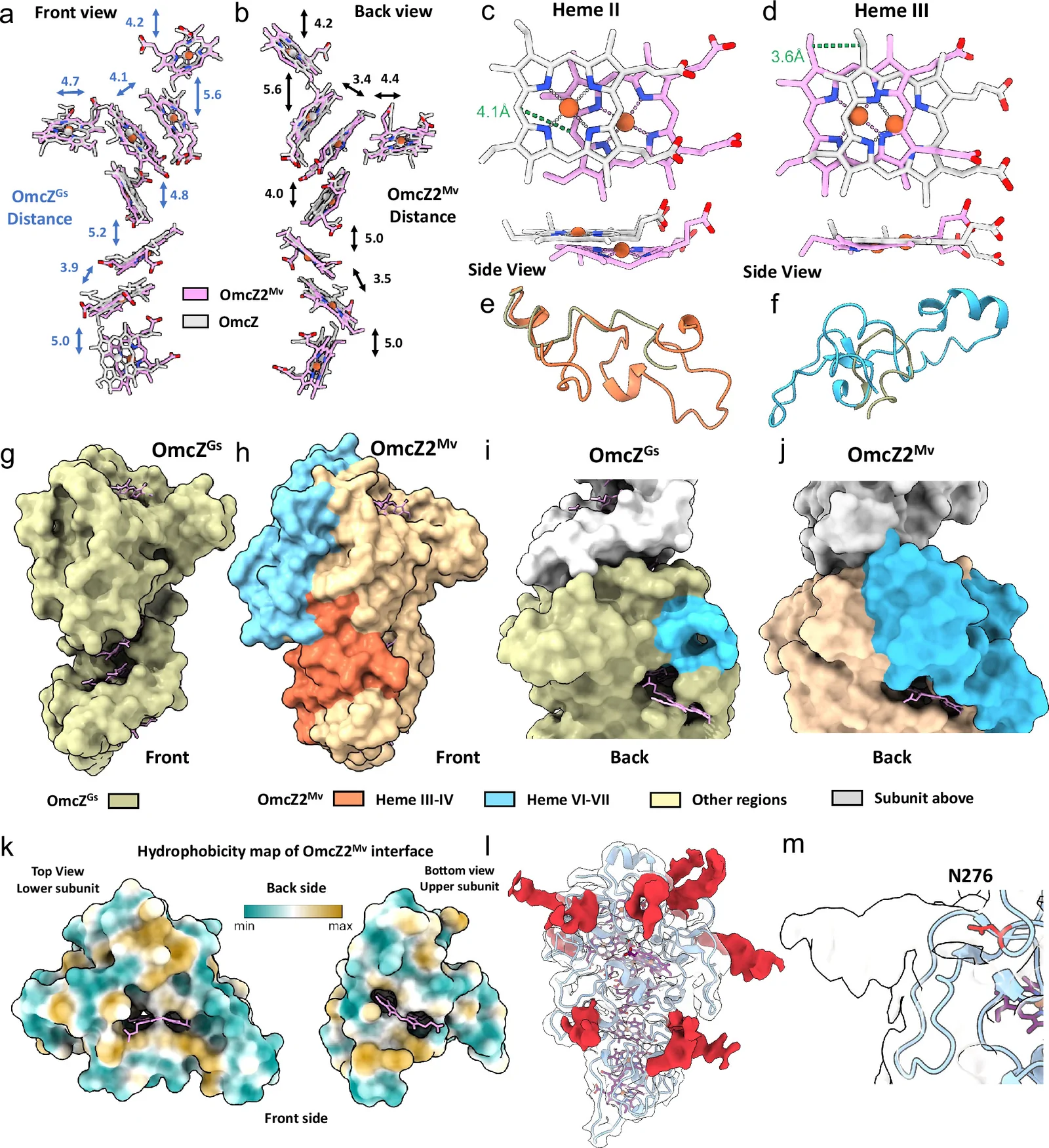
Structural features of OmcZ2^Mv^ nanowires optimized for high electron conductivity. **a** Front and **b** back views of the superimposed heme-chain of OmcZ^Gs^ (PDB 7LQ5; gray hemes, blue arrows) and OmcZ2^Mv^ (pink hemes, black arrows) with edge-to-edge heme distances labeled in Å. **c** Heme II and **d** Heme III exhibit displacement and non-planar conformations in OmcZ2^Mv^ relative to OmcZ^Gs^. Expanded inter-heme regions between **e** Heme III–IV and **f** Heme VI–VII in OmcZ2^Mv^ (orange in **e** and blue in **f**) compared to OmcZ^Gs^ (olive). **g**, **h** Front and **i**, **j** back views of **g** OmcZ^Gs^ protomers showing solvent-exposed Heme I and III, and **h** OmcZ2^Mv^ showing expanded regions of Hemes III–IV (orange) and VI–VII (blue). The interfacial area and the depth of the heme pocket are lower in **i** OmcZ^Gs^ than in **j** OmcZ2^Mv^. **k** Top and bottom views of the archaeal protomer interface showing hydrophilic (cyan) and hydrophobic (gold) regions. **l** OmcZ2^Mv^ shows extra densities (red) at 10 asparagine residues as **m** exemplified by N276 in red, consistent with N-glycosylation.

The spacing and orientation of hemes are critical for efficient EET within conductive filaments. Compared with OmcS nanowires, OmcZ^Gs^ exhibits shorter inter-heme distances and consequently a nearly 1000-fold higher electron conductivity^26,27,30^. Heme packing in OmcZ2^Mv^ appears to be further optimized for EET compared to OmcZ^Gs^ in three ways. Firstly, several hemes in OmcZ2^Mv^ are displaced compared to OmcZ^Gs^ (Fig. 4a, b; Supplementary Fig. 7A–F), especially at the bottom part of the protomer where Heme II and III are shifted by about 4 Å (Fig. 4c, d). Secondly, some hemes are not in a planar conformation, which impacts their redox potential and nanowire conductivity^43^ (Fig. 4c, d; Supplementary Fig. 7A–F). Thirdly, OmcZ2^Mv^ has reduced edge-to-edge distances of hemes compared to OmcZ^Gs^ (Fig. 4a, b, Supplementary Table 4). Together, these structural features might be optimizations for high conductivity in OmcZ2^Mv^ nanowires, potentially enabling efficient long-range EET.

OmcZ2^Mv^ possesses an expanded protein scaffold, with ~30% more residues than OmcZ^Gs^ (Supplementary Tables 3, 5), primarily in the regions between binding sites of Heme III and IV, and Heme VI and VII (Fig. 4e, f). These expansions substantially alter the solvent exposure of hemes. In OmcZ^Gs^, Heme I and III, and the branched Heme VI are largely solvent exposed, whereas in OmcZ2^Mv^ these hemes are buried within pockets formed by the expanded loops (Fig. 4g–j). This heme insulation potentially reduces electrical current leakage and nanowire aggregation by preventing hydrophobic interactions between exposed hemes. Furthermore, the expanded loop between Heme VI and VII increases the interfacial area between adjacent protomers from ~1200 Å² to ~1400 Å², stabilizing inter-subunit interactions and increasing filament stability (Fig. 4j).

Consistent with its shorter edge-to-edge heme distances, OmcZ2^Mv^ exhibits a denser network of salt bridges and cation-π interactions than OmcZ^Gs^. These include the connection of Heme III with Heme I and IV via a salt bridge to K117 (Supplementary Fig. 8A). The expanded region between Heme III and IV not only covers Heme I and III but also stabilizes them through salt bridges between the heme propionate groups and R105 (Supplementary Fig. 8B). Furthermore, Heme IV, VII and VIII are connected by salt bridge interactions between D145 close to Heme IV and R287 in the α-helix connecting Heme VII and VIII (Supplementary Fig. 8C). At the same time Heme VI is stabilized by interactions with K258 (Supplementary Fig. 8D). These heme-stabilizing interactions tightly hold the heme chain and the protein shell together, distinct from the salt bridges K105-D189 and D120-K108 that stabilize Heme IV in OmcZ^Gs^ (Supplementary Fig. 8G, H). Besides, other salt bridges and cation-π interactions help connect separate loops and maintain structural integrity of OmcZ2^Mv^ (Supplementary Fig. 8E, F). No specific salt bridges or cation-π interactions are observed at the protomer interfaces. Because hydrophobic pads are located on both sides of the interface (Fig. 4K) and heme is a hydrophobic molecule, the major driving force for the interfacial interactions should come from the hydrophobic effect and the stacking force between the heme pair across the interface.

Finally, OmcZ2^Mv^ displays extensive additional electron densities at 10 of the 11 N-glycosylation motifs (NXS/T (X ≠ P)) (Fig. 4l, m and Supplementary Fig. 9A), whereas OmcZ^Gs^ contains only three such motifs and shows no detectable extra densities (Supplementary Fig. 9B)^27,28^. These putative protruding N-glycans in OmcZ2^Mv^, extending up to ~20 Å from the filament surface, are enriched near turns and hydrophobic regions (Supplementary Fig. 9D), possibly preventing filament aggregation or facilitating adsorption to surfaces. Additionally, these putative glycosylations, which add 1–3 kDa per glycosylation site, could account for the additional apparent molecular weight of ~30 kDa observed for OmcZ2^Mv^ on gels (Fig. 3c).

### OmcZ-family proteins share key structural features

**Fig. 5:**
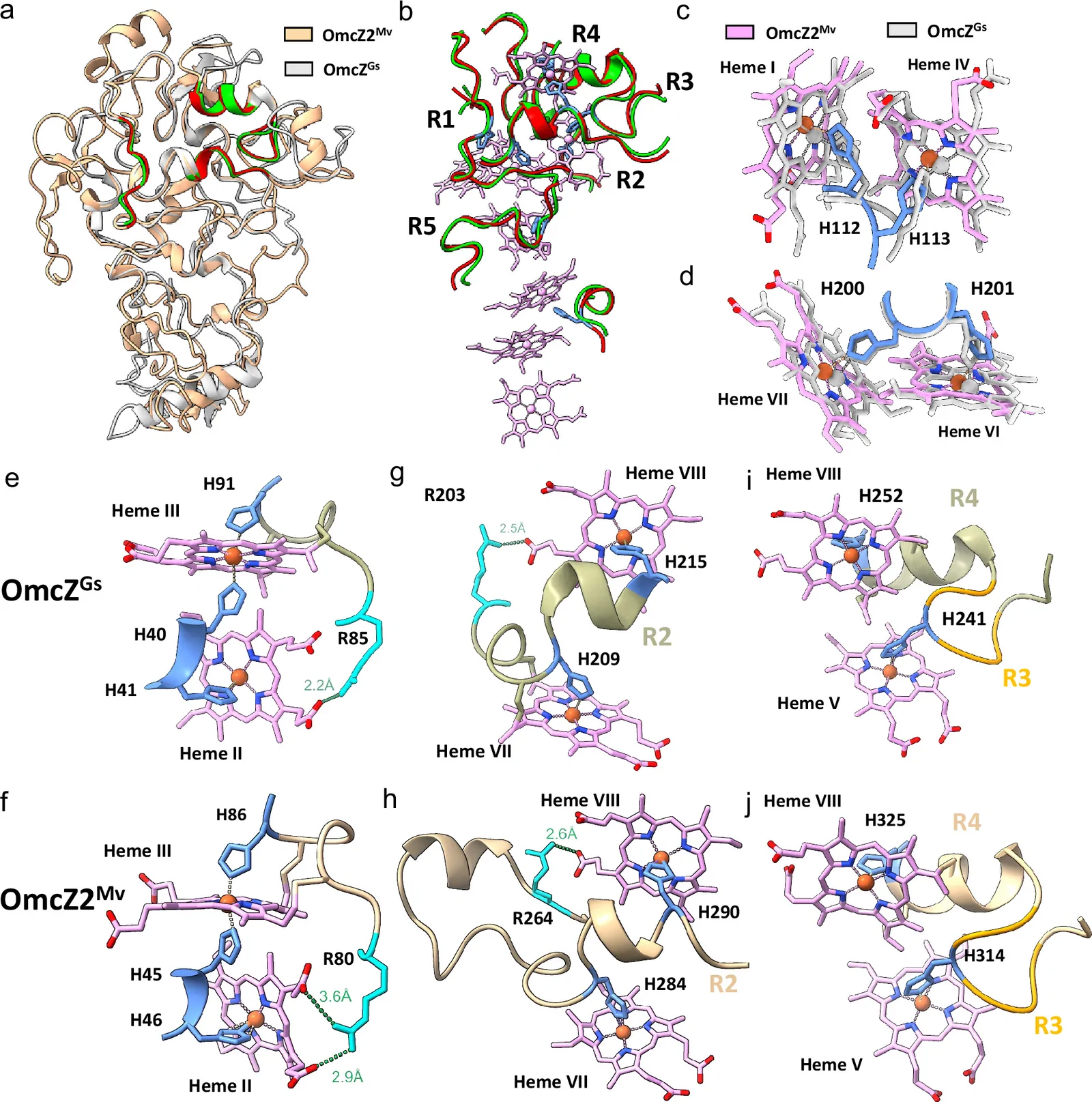
Conserved structural features of OmcZ-family nanowires. **a** Superimposed structures of OmcZ^Gs^ (PDB 7LQ5; gray/green) and OmcZ2^Mv^ (beige/red) protomers showing conserved regions in green/red, and **b** corresponding structurally conserved regions (R1–R4) with R5 marking an ascending lasso-shaped loop. Conserved consecutive histidines (blue) linking **c** adjacent Hemes I and IV and **d** Hemes VI and VII in OmcZ^Gs^ (gray) and OmcZ2^Mv^ (pink). Comparison of OmcZ^Gs^ (top row) and OmcZ2^Mv^ (bottom row) showing linkages of **e**, **f** Heme II and III via consecutive histidines and stabilizing arginine-propionate salt bridges, **g**, **h** Heme VII and VIII via a short α-helix bearing heme coordinating histidines and a propionate salt bridge, and **i**, **j** Heme V and VIII via the conserved region R4, a short α-helix bearing heme coordination histidines, and the conserved region R3 in orange.

Despite originating from different domains of life and sharing low sequence identity, bacterial OmcZ^Gs^ and archaeal OmcZ2^Mv^ share a highly conserved molecular architecture. Aligning 90 OmcZ-family proteins from both bacteria and archaea revealed four highly-conserved short sequence regions that form a “core quadrangle” surrounding Heme V and Heme VIII (Fig. 5a): Region 1 (DGHYV/IP, residues 149-154), Region 2 (L/IHNIE/Q, residues 289-293), Region 3 (GXGHI/LG, residues 311–316), and Region 4 (DCXGCH, residues 320-325).

Furthermore, we identified a spatially conserved set of 91 residues, encompassing these four regions, with a root-mean-square deviation of 0.98 Å (Fig. 5b). These residues cluster predominantly on the upper half of the protomer and include multiple histidine residues coordinating Heme I, IV, V, VII, and VIII (Fig. 5b). Notably, the regions surrounding the triangular arrangement of Heme V, VII, and VIII display the highest degree of structural conservation, underscoring their central role in maintaining the specific architecture of OmcZ-family proteins.

Because loops and turns dominate OmcZ-family proteins, only a few conserved structural features are responsible for organizing the heme chain and maintaining nanowire integrity. Central to this organization are three conserved histidine-histidine (HH) motifs that link adjacent heme pairs in both OmcZ2^Mv^ and OmcZ^Gs^ together. Specifically, H112-H113 (H96-H97 in OmcZ^Gs^) coordinates Hemes I and IV (Fig. 5c), H200-H201 (H182-H183) coordinates Hemes VI and VII (Fig. 5d), and H45-H46 (H40-H41) coordinates Hemes II and III (Fig. 5e, f).

Additional interactions further stabilize these heme pairs. For Heme II and III, the HH motif is complemented by salt bridges between the propionate groups of Heme II and R80 (R85), located immediately upstream of the Heme III binding motif (**R**NCHDCH) (Fig. 5e, f). A similar dual-sided stabilization is observed for the Heme VII-VIII pair. On one side, Heme VII is linked to Heme VIII via a short α-helix bearing histidines H284 (H209) and H290 (H215) at opposite ends. On the other side, a salt bridge is formed between the propionate group of Heme VIII and R264 (R203), further stabilizing this heme pair (Fig. 5g, h). Heme VIII is also structurally coupled to Heme V through an α-helix on the conserved Region 4 that contains the Heme VIII-binding motif, while the Heme V-coordinating histidine H314 (H241) on the conserved Region 3 precedes this α-helix (Fig. 5i, j). Together, these interactions position Heme VII as a central point within the heme chain: Heme VII adopts an approximately parallel orientation relative to Hemes IV, V, and VI, and is connected to adjacent hemes through both HH motifs and α-helical linkers.

AlphaFold predictions of full-length OmcZ proteins indicate that the cleaved C-terminal regions adopt β-sandwich folds resembling immunoglobulin-like domains, with three such domains in OmcZ2^Mv^ and two in OmcZ^Gs^ (Supplementary Fig. 10A, B). In both proteins, the cleavage sites are located in a loop separating the nanowire-forming N-terminal region from the C-terminal region containing β-sandwich folds (Supplementary Fig. 10C, D). These sites follow bulky aromatic residues and are sandwiched by small amino acids, indicating a general mechanism for the maturation of OmcZ-type nanowires^27,44^.

### OmcZ2^Mv^ presents a broad distribution in ANME archaea and Borgs

Archaeal OmcZ homologs are predominantly found in ANME archaea, determined from analysis of the NCBI ClusteredNR database. A systematic analysis of 212 MAGs of ANME archaea (Supplementary Fig. 11A) revealed that OmcZ homologs are widespread across the ANME-2 clade with a prevalence of 20–70% within the subclades 2a-d, but occur rarely in ANME-1 and are absent from ANME-3 MAGs (Supplementary Fig. 11B). Notably, gene copy number expansion of OmcZ was observed exclusively in ANME-2d, the freshwater lineage represented by the genus “*Ca*. Methanoperedens”, whereas ANME-2abc lineages only encode a single copy (Supplementary Fig. 11C). Similar gene expansions were also observed in Borg elements associated with “*Ca*. Methanoperedens” (Supplementary Fig. 11C), expanding previous reports of OmcZ homologs on Borg elements^31,32^.

**Fig. 6:**
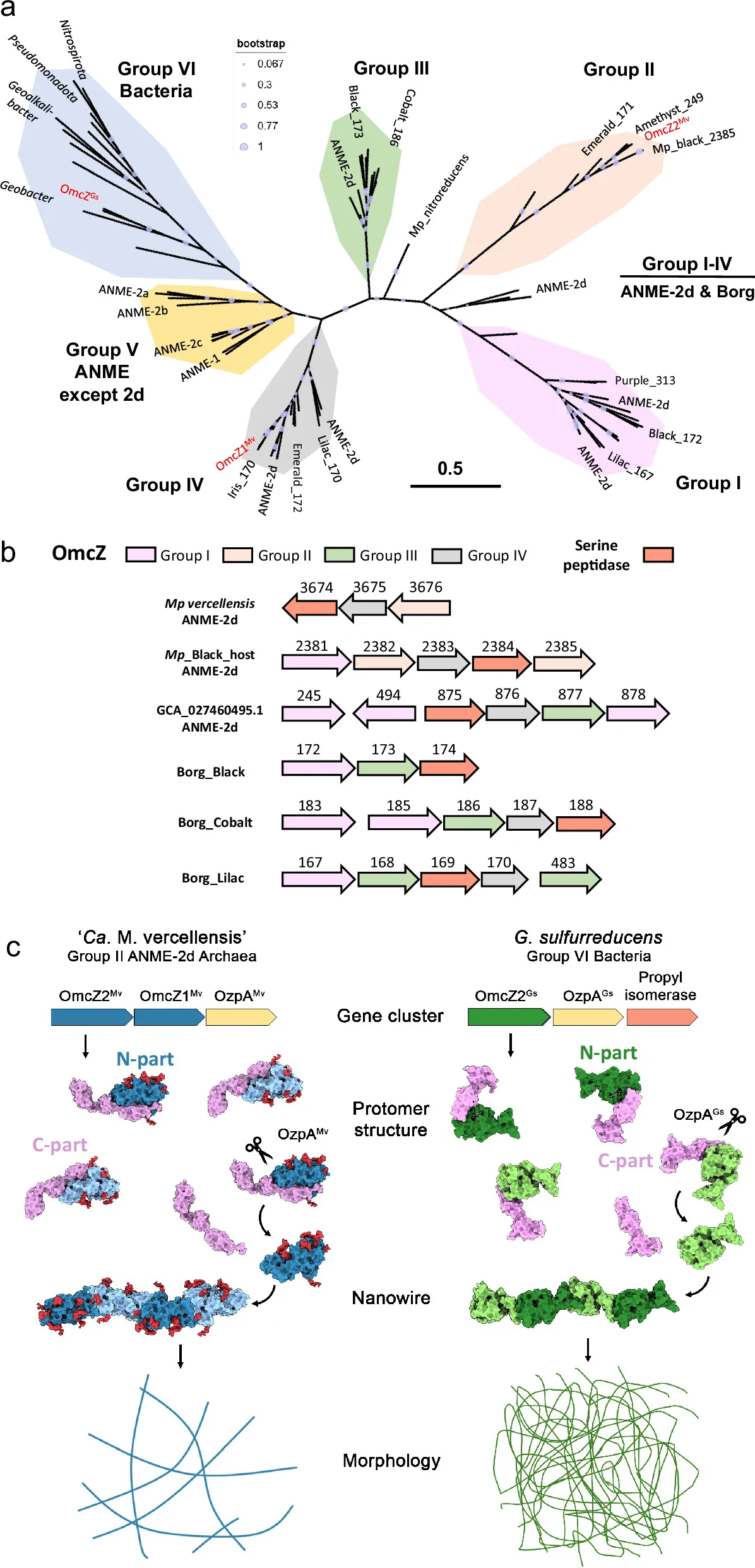
Distribution of OmcZ homologs across bacteria, archaea and Borg elements, and schematic comparison of key features of archaeal and bacterial OmcZ homologs. **a** Phylogenetic classification of the filament-forming domains of OmcZ nanowires into six major groups. Groups I–IV comprise ANME-2d archaea (“*Ca*. Methanoperedens”) and Borg elements, Group V comprises other ANME lineages, and Group VI comprises bacteria. Borg elements are named after colors, and *Mp* denotes “*Ca*. Methanoperedens” species. OmcZ proteins discussed in this study are highlighted in red. Circle size reflects bootstrap values. **b** Genomic organization of OmcZ homologs in ANME-2d and associated Borg elements, illustrating the frequent co-occurrence of multiple OmcZ homologs from different phylogenetic groups. Gene identifiers are indicated above each arrow. **c** Gene clusters of “*Ca*. M. vercellensis” OmcZ2^Mv^ (left) and *G. sulfurreducens* OmcZ^Gs^ (right) are displayed. The N-terminal part of OmcZ2^Mv^ (blue) exhibits extensive surface modifications (red) that are absent in OmcZ^Gs^ (green). Protomers assemble into nanowires following cleavage of the C-terminal part (pink) by OzpA. Mature OmcZ2^Mv^ nanowires display less aggregation than their OmcZ^Gs^ counterparts.

By including bacterial homologs in the dataset, OmcZ-family proteins could be classified into six major phylogenetic groups (Fig. 6a). Groups I-IV consist of OmcZ homologs found in ANME-2d and Borg elements only, Group V comprises ANME-1 and ANME-2abc lineages, and Group VI contains bacterial OmcZ homologs (Fig. 6a and Supplementary Fig. 12). The OmcZ2^Mv^ nanowire clusters most closely with Group II, sharing 82.1%, 62.3%, and 62.6% protein sequence identity with Borg Amethyst_249, Borg Emerald_171, and the “*Ca*. Methanoperedens” host of Borg Black_172, respectively (Fig. 6a and Supplementary Fig. 12). In contrast, OmcZ2^Mv^ is evolutionarily distant from the bacterial OmcZ^Gs^, which belongs to Group VI, as well as from OmcZ1^Mv^, the second OmcZ homolog encoded by “*Ca*. M. vercellensis”, which falls within Group IV (Fig. 6a and Supplementary Fig. 12).

ANME-2d archaea and Borg elements frequently encode multiple OmcZ homologs from different phylogenetic groups (Fig. 6b and Supplementary Fig. 11D), suggesting a differential use of OmcZ variants under different environmental or metabolic conditions. Archaeal OmcZ gene clusters commonly co-occur with serine peptidases (Fig. 6b and Supplementary Fig. 11D), consistent with a role analogous to *Geobacter* OmcZ-cleaving protease OzpA that facilitates protomer polymerization^27,33^.

The OmcZ groups also differ markedly in protein size and glycosylation potential. OmcZ homologs from Groups I-II consist of substantially more amino acid residues than the other groups (Supplementary Fig. 11E). Additionally, OmcZ proteins from ANME-2d and Borg elements exhibit the highest percentage of N-glycosylation motifs, exceeding that of OmcZ^Gs^ by more than fivefold (Supplementary Fig. 11F). For example, Group I OmcZ from Borg Amber_301 encodes 16 motifs across 372 residues making up ~12% of the protein (Supplementary Fig. 9C), indicating that N-glycosylation may represent a specific evolutionary adaptation.

Together, this analysis supports the widespread distribution and diversification of OmcZ homologs across ANME lineages, with OmcZ2^Mv^ nanowires representing a structural framework for EET in anaerobic methanotrophs. Moreover, its close evolutionary relationship with Borg-encoded OmcZ proteins provides new insight into the potential physiological roles of these enigmatic extrachromosomal elements.

### Etymology

“*Candidatus* Methanoperedens vercellensis” sp. nov. *vercellensis* (ver.cel.len’sis. L. masc. adj. *vercellensis*, pertaining to Vercelli, Italy, the site from which the organism was initially enriched).

## Discussion

This study establishes “*Ca*. Methanoperedens vercellensis” (formerly “*Ca*. Methanoperedens Vercelli Strain 1”) as a near-axenic model organism for investigating electrode-associated growth and EET in anaerobic methanotrophic archaea. EET-performing cells predominantly produced OmcZ2^Mv^ nanowires, for which we determined a 3.3 Å-resolution cryo-EM structure revealing features that could help explain high electron conductivity and efficient EET.

Within 119 days of incubation in a BES, the relative abundance of “*Ca*. M. vercellensis” increased from 9.7% in the inoculum to 95.8%, resulting in a near-axenic culture. To our knowledge, such a high level of enrichment has not been reported previously and highlights the strong selective advantage of electrode-associated growth together with a mix of bacteria-suppressing antibiotics. Furthermore, the absence of soluble electron acceptors such as nitrate eliminated the need for facultative syntrophic partners, such as denitrifying anaerobic methane oxidizing bacteria or anaerobic ammonium oxidizing bacteria, to mitigate toxic nitrite accumulation^45,46^.

We demonstrated that BESs provide a powerful platform to study ANME physiology by deriving current-based doubling times of 8.1 ± 1.1 days at 22.2 °C, which shortened to 5.5 ± 0.9 days at 30 °C. This temperature-dependent increase in growth rate corresponds to an apparent Q10 of 1.6. This doubling time of “*Ca*. M. vercellensis” is less than half of the previously measured doubling times for Methanoperedenaceae (19 days at 22 °C)^13^, and substantially shorter than reported values for marine ANME species^12,14,15^.

Electrode-grown “*Ca*. M. vercellensis” specifically oxidizes methane, while failing to convert ethane into current, in alignment with earlier observations that the methane-activating enzyme Methyl-Coenzyme M Reductase (MCR) lacks internal channels that would allow diffusion of larger alkanes into the active site^42^. The rapid recovery of current after replacing ethane with methane further indicates that ethane is not inhibitory, contrasting with previous hypotheses that non-methane hydrocarbons inhibit ANME archaea^47^.

The detection of menaquinone as the membrane-integral electron carrier in “*Ca*. M. vercellensis” is consistent with earlier work^40^ and highlights a fundamental metabolic divergence among ANME clades. Freshwater ANME-2d encode the futalosine pathway for menaquinone biosynthesis, whereas their marine relatives, ANME-2abc and ANME-3, lack this pathway and are instead thought to rely on methanophenazine^16^. This distinction is notable given the substantial difference in redox potential between menaquinone and methanophenazine (E^0^’ = −80 mV and −165 mV for menaquinone-8 and methanophenazine, respectively).

Prolonged electrode-associated growth markedly altered the respiratory physiology of “*Ca*. M. vercellensis”, which did not resume nitrate-dependent methane oxidation within 1 week, despite the absence of genomic changes, suggesting a tight regulatory control of nitrate reductase expression rather than genetic loss. In contrast, methane oxidation readily occurred in the presence of ferrihydrite, indicating that the MHCs facilitating EET to the electrode can also mediate electron transfer to insoluble metal oxides. This apparent overlap in electron transfer capability contrasts with transcriptomic data from enrichment cultures of “*Candidatus* Methanoperedens nitroreducens”, where distinct subsets of MHCs were expressed during iron reduction versus electrode respiration^22^.

Here, we report the structure of archaeal OmcZ nanowires at 3.3 Å resolution obtained by native purification under physiologically relevant conditions. In contrast, previously reported structures of bacterial OmcZ^Gs^ nanowires were solved in highly alkaline pH 10.5 ethanolamine buffers^27,28^, conditions known to induce conformational rearrangements in *c*-type cytochromes^48^. Moreover, earlier OmcZ nanowire structures were resolved at 3.5 and 4.0 Å resolution, limiting the visualization of fine structural features^27,28^. Comparison of OmcZ2^Mv^ with OmcZ^Gs^ revealed three major distinctions in the organization of the heme chain, the nanowire surface architecture, and the morphology of filaments.

First, the heme chain in OmcZ2^Mv^ exhibits shorter edge-to-edge distances than OmcZ^Gs^, a feature that exponentially enhances electron transfer rates^49^. Previous studies on OmcS and OmcZ nanowires have shown that more compact heme arrangements can increase electrical conductivity by up to three orders of magnitude^26,30^. By analogy, the reduced inter-heme spacing in OmcZ2^Mv^ possibly supports higher electrical conductivity than in OmcZ^Gs^. Additionally, OmcZ2^Mv^ nanowires exhibit non-planar heme conformations. Apart from shortening inter-heme distances, such heme distortions, which are common among cytochromes, are known to modulate redox potentials, thereby lowering energetic barriers for electron transfer^50–52^. The non-planar heme geometries in OmcZ2^Mv^ likely arise from structural constraints imposed by the heme-binding motifs, together with stabilizing salt bridges to heme propionate groups. Moreover, shifted heme propionate groups, like those observed in OmcZ2^Mv^, have been associated with increased heme stability^53^.

Second, the heme chain in OmcZ2^Mv^ nanowires is more insulated than in OmcZ^Gs^. In OmcZ^Gs^, solvent-exposed hemes can promote nanowire aggregation and facilitate inter-nanowire electron transfer. In contrast, the expanded inter-heme regions in OmcZ2^Mv^ shield the heme chain, likely reducing current leakage, and limiting filament aggregation and flexibility. This insulation is further enhanced by large extra densities observed on ten surface-exposed asparagine residues within N-glycosylation motifs (Fig. 6). Similar densities have been reported for OmcE and ECN^Av^ nanowires^23,29^, but in much lower abundance and without defined motifs.

N-glycosylation is a common feature of archaeal proteins and may serve several stabilizing and organizational roles in OmcZ2^Mv^ nanowires^54,55^. Notably, eight out of the ten modified asparagines on OmcZ2^Mv^ are located on turns, and four follow aromatic residues, a context previously shown to enhance protein stability^56^, potentially compensating for the missing prolyl isomerase in the OmcZ gene cluster (Fig. 6). Moreover, because most N-glycosylation motifs are positioned near hydrophobic regions and hydrophobic interactions dominate protomer interfaces, these putative modifications may suppress nonspecific protomer-protomer contacts while favoring controlled, end-to-end self-assembly. The large size of the protruding densities could further act as barriers that prevent filament bundling, analogous to glycosylation-mediated archaellum organization in *Halobacterium salinarum*^57^. Finally, because OmcZ-type nanowires must associate with the cell surface to enable electron transfer, the putative N-glycosylation may also facilitate anchoring of OmcZ2^Mv^ nanowires to the archaeal cell envelope.

Third, the nanowire morphology of OmcZ^Gs^ and OmcZ2^Mv^ differs substantially. Whereas OmcZ^Gs^ nanowires aggregate extensively and densely fill the extracellular space between bacterial cells and electrodes, OmcZ2^Mv^ forms straighter, less densely packed filaments (Fig. 6). Given the relatively slower growth of “*Ca*. M. vercellensis” compared to *G. sulfurreducens*, evolutionary selection may favor a more efficient nanowire architecture that supports long-range electron transfer without extensive aggregation. In *G. sulfurreducens*, this role is fulfilled by straight, conductive OmcS nanowires. However, OmcS-type nanowires and their accessory genes are absent in archaea^58^. It could therefore be possible that OmcZ2^Mv^ nanowires have convergently evolved morphological and functional features resembling those of bacterial OmcS nanowires.

Despite several distinctions between OmcZ2^Mv^ and OmcZ^Gs^, key design principles are conserved across the OmcZ family, even at low (~30%) sequence identity. The OmcZ-family proteins exhibit a remarkably conserved molecular architecture centered on a “core quadrangle” surrounding hemes V and VIII, which represents the most invariant region across all analyzed homologs. Structural alignment revealed that this conserved core anchors the organization of the heme chain and reinforces nanowire integrity. In the absence of extensive secondary structures, heme positioning and connectivity are maintained by a limited set of conserved motifs, most notably three pairs of consecutive histidine residues that coordinate adjacent heme pairs, reinforced by arginine-mediated salt bridges to heme propionate groups. Together, these conserved interactions position Heme VII as a central hub within the chain and highlight how OmcZ-family nanowires preserve a shared conductive architecture despite sequence divergence.

A systematic phylogenetic analysis across ANME archaea revealed that OmcZ homologs are widespread among ANME-2 lineages, but rare in ANME-1 and absent in ANME-3. These homologs cluster into six distinct phylogenetic groups that differ in protein length and number of glycosylation motifs (Supplementary Fig. 11B). Notably, only freshwater ANME-2d, represented by the family Methanoperedenaceae, harbor multiple OmcZ homologs within a single genome. This pattern suggests that OmcZ gene expansion may contribute to niche adaptation and enhanced metabolic flexibility in ANME-2d. Although “*Ca*. M. vercellensis” encodes a large plasmid without OmcZ homologs, OmcZ genes are frequently found on giant (>1 Mb) extrachromosomal elements known as Borgs that are associated with other “*Ca*. Methanoperedens” genomes^31,32^. Borgs encode numerous metabolic genes linked to methane oxidation that could augment host metabolism. Supporting this notion, a Borg-encoded OmcZ homolog was found to be expressed at higher levels than their chromosomal counterparts^32^. Together, these observations support the notion that maintaining multiple OmcZ variant from distinct phylogenetic groups is advantageous for Methanoperedenaceae.

Beyond freshwater ANME-2d, cytochrome genes were upregulated when marine ANME lineages couple methane oxidation to sulfate reduction through DIET with SRB via conductive cytochrome nanowires^6,7^. Although the molecular identity of these nanowires remains unresolved, marine ANME-2abc frequently encode and express OmcZ homologs (Supplementary Fig. 11B)^16,59^. This widespread presence raises the intriguing possibility that, in addition to long-range EET to electrodes described here, OmcZ-type nanowires also facilitate DIET to SRB partners. Such nanowire-mediated DIET could help explain the rapid metabolism without diffusible intermediates^6^ and filamentous structures^7^ reported in prior studies.

Our study has several limitations. Although we obtained a near-axenic electrode-grown culture of “*Ca*. M. vercellensis”, low-abundance community members remained present and may still influence its physiology during electrode-associated growth. Moreover, the relatively slow growth rates of this archaeon, combined with the limited scale of the BESs, restricted the amount of biomass available and prevented extensive biochemical and physicochemical characterization of the nanowires. While additional nanowire candidates were observed by negative-stain TEM and detected by mass spectrometry, insufficient particle numbers precluded high-resolution cryo-EM structure determination. Furthermore, the chemical identity of the extra densities observed on surface-exposed asparagine residues could not be directly verified, leaving the proposed N-glycosylation tentative. Finally, the lack of established genetic tools for “*Ca*. M. vercellensis” currently prevents direct genetic investigation of OmcZ2^Mv^ nanowire function or systematic testing of the physiological roles of specific structural features and surface modifications.

## Methods

### Cultivation of “*Ca*. Methanoperedens vercellensis” in bioelectrochemical systems

BES were inoculated using a bioreactor enrichment culture originally sampled from a rice paddy field in Vercelli, Italy, and operated under continuous methane supply and nitrate-limiting conditions since March 2014^60^, with adaptations to the bioreactor operations described in ref. ^42^.

“*Ca*. M. vercellensis” was cultivated in a two-chamber, jacketed BES (Adams & Chittenden Scientific Glass, CA, USA) as described before^20,21^, with the change of anode material from gold to graphite felt (13.8 cm^2^, 4.7 mm thickness, 0.107 g cm^-3^ density, G475A Soft Graphite Battery Felt, AvCarb Material Solution, MA, USA). The anode potential of BESs was controlled by an EmStat4 LR potentiostat (PalmSens BV, Houten, NL) at 0 V vs. standard hydrogen electrode (SHE) using a 3 M Ag/AgCl reference electrode (Prosense, Oosterhout, NL); current data were collected using the MultiTrace 4 software. In an anaerobic hood (97% N_2_, 3% H_2_), bioreactor biomass (60 mL, 0.13 g dry weight) was washed tree times in BES medium, before inoculation of the anode chamber. The medium was amended from previous reports^20,21^ and composed of per liter 0.2 g MgCl_2_.6H_2_O, 0.27 g CaCl_2_.2H_2_O, 0.1 g KH_2_PO_4_, 0.5 g NH_4_Cl, 2.384 g HEPES (4-(2-hydroxyethyl)-1-piperazineethanesulfonic acid), 0.1 mL Wolin’s vitamin solution (DSMZ 141), 0.5 mL trace element solution. The trace element solution contained per liter 0.12 g CeCl_3_.7H_2_O, 0.6 g CoCl_2_.6H_2_O, 4.0 g CuSO_4_, 0.07 g H_3_BO_3_, 1.0 g MnCl_2_.4H_2_O, 0.35 g Na_3_MoO_4_.2H_2_O, 0.1 g Na_2_WO_4_.2H_2_O, 0.95 g NiCl_2_.6H_2_O, 0.14 g SeO_2_, 1.44 g ZnSO_4_.7H_2_O. The medium was adjusted to pH 7.8 with NaOH, filtered (0.2 µm pore size), and made anoxic by gas vacuum cycles with Ar/CO_2_ (95:5), after which the pH stabilized at ~7.3. To the anoxic medium, 1 mL of anoxic 10 mM FeCl_2_ HCl (pH 2.5) was added. After 1 week, residual biomass that had not formed a biofilm on the electrode was removed in a medium exchange. To suppress bacterial growth, streptomycin sulfate, vancomycin HCl, ampicillin sodium salt, and kanamycin sulfate (all at 50 μg mL^−1^) to which “*Ca*. Methanoperedens sp.” BLZ2 was previously shown to be resistant^61^, were added after 16 days and regularly during operation as indicated in the figures and the text.

BES pH was manually maintained between 6.9 and 7.4 using an anoxic KOH solution. When daily pH adjustment became necessary, one-quarter of the electrode biofilm was cut into ~0.25 cm^2^ pieces and used to inoculate a clean BES. Biomass transfer occurred through collisions between electrode pieces in the suspension and the new wired electrode. After 2–7 days, the old electrode pieces were removed, and the medium was refreshed. In total, five duplicate batch incubations were performed: one initial inoculation from a bioreactor, followed by four sequential transfers (Figs. 1, S1).

### Methane-dependent current

To determine the percentage of methane-dependent current, the continuous flow of CH_4_/CO_2_ (95:5, 10 mL min^−1^) was switched to Ar/CO_2_ (95:5, 40 mL min^−1^) for approximately 6 h. Methane-dependent current was calculated as the percentage of current loss after 6 h of flushing with Ar/CO_2_ relative to the current immediately before the measurement. Similarly, to assess whether “*Ca*. M. vercellensis” can oxidize ethane, the continuous methane supply was exchanged for argon to strip residual methane overnight. Continuous flushing was stopped, and 20 mL of ethane was added to the headspace for 3 days. After 3 days, regular sparging with CH_4_/CO_2_ was resumed.

### Doubling time calculations

The doubling time of “*Ca*. M. vercellensis” enriched biofilms was extrapolated based on the doubling time of the current. Current data (recorded every minute for at least 2 days) was averaged for every 60 datapoints and fitted to an exponential growth equation (Eq. 1):

1 $$\left(\right.A\left(t\right)=A\left(0\right){e}^{\mu t}$$

Where *A* is the current, *t* is time and *μ* is the growth rate.

### Coulombic efficiency

The coulombic efficiency of BESs was determined during batch 4 by switching from continuous methane flushing to flushing with Ar/CO2 (95:5, 40 mL min^−1^) overnight. Then, the BESs were closed, and 20 mL of ^13^C-CH_4_ was injected into each BES. Headspace samples (50 µL) were analyzed four times in 96 h using a gas chromatograph coupled to a flame ionization detector and mass spectrometer (GC-FID-MS, Agilent 8890 GC System, and Agilent 5977B GC/MSD, Agilent Technologies, Santa Clara, CA, USA). Methane consumption was sufficiently high to be detected solely from the FID signal. Headspace methane concentrations were calculated using the ideal gas law, and methane consumption rates were determined by linear regression of four data points per BES. Current data (measured every minute) were averaged and converted from µA to nmol h^−1^ using the elementary charge (Coulomb) and Avogadro’s constant. Coulombic efficiency was expressed as the ratio of current to methane consumption rate.

### DNA extraction, metagenome sequencing and analysis

DNA was extracted from electrode biofilms from batch 1–3 following the Powersoil DNeasy kit protocol (Qiagen, Hilden, Germany) with the addition of a 10 min bead beating step (50 s^−1^, Tissuelyser LT, Qiagen, Hilden, Germany) on small cut-out pieces of up to 1/8th of electrode. DNA was eluted in autoclaved UV-sterilized water. Short read whole genome sequencing (Illumina) of a bioreactor sample and after BES batch 1 were sequenced in-house: 1 ng of DNA was used for Illumina library preparation using the Nextera XT Library Preparation Kit (Illumina, San Diego, California, USA). Libraries were checked for quality and size distribution using the Agilent 2100 Bioanalyzer and the High sensitivity DNA kit (Agilent Technologies, Santa Clara, California, USA). Quantitation was performed using the Qubit dsDNA HS Assay Kit (Thermo Fisher Scientific Inc Waltham, USA). Libraries were normalized, pooled and sequenced (2x300bp) using an Illumina MiSeq (Illumina) and the MiSeq Reagent Kit v3 according to the manufacturer’s protocol^61^. After BES batch 2 and 3, short read sequencing was carried out by Macrogen Europe (Amsterdam, NL), where libraries were prepared using TruSeq Nano DNA Kit, which were sequenced using NovaSeqX 150 bp paired-end. Additionally, two DNA samples were used for in-house Oxford Nanopore Technologies (ONT) sequencing. ONT sequencing was done with ~400 ng DNA after library preparation using the Native Barcoding Kit (SQK-NBD114.24) according to the manufacturer’s protocols (Oxford Nanopore Technologies, Oxford, UK). The libraries were loaded on a Flow Cell (FLO-MIN114, R10.4.1) and sequenced on a MinION Mk1C device (Oxford Nanopore Technologies, Oxford, UK) according to the manufacturer’s instructions. Basecalling was performed on the fly with MinKNOW 24.02.16 on the Mk1C device using the Dorado 7.3.11 with the fast 400 bps model, both provided by ONT.

Nanopore long reads were quality-filtered using Filtlong v0.2.1 (https://github.com/rrwick/Filtlong) using the parameters -min_length 1000 and -keep_percent 90. Filtered reads were assembled with Flye v2.8.3^62^ with the -meta setting (metaFlye), resulting in three circularized MAGs. These were polished with one round of racon^63^ v1.4.21 and three rounds of pilon5 v1.2.4^64^. To complement both long and short read data, Aviary v0.9.0 (https://github.com/rhysnewell/aviary) was used for hybrid assembly and binning of all obtained short- and long-reads from the BESs after batch 2 and 3 (four and two read-sets, respectively); Aviary integrates several different assemblers and binning tools. Resulting MAGs were merged with the circularized genomes and dereplicated with dRep v3.4.2^65^ using a threshold of >40% completeness and <10% contamination as estimated by CheckM2^66^. In parallel, short-read DNA obtained from the inoculum was assembled and binned separately with Aviary. Resulting MAGs were dereplicated together with MAGs obtained from the BESs using the same criteria. Relative abundances were obtained from remapping of trimmed Illumina reads on this non-redundant genome set using CoverM v0.7.0^67^. Phylogenetic classification of the bacterial and archaeal genomes was assigned using GTDB-Tk v2.4.0 (release R220) using the classify_wf command^68^. Genomes were annotated using Prokka v1.14.6^69^ and EggNOG v5.0 with eggNOG-mapper v2.1.12^70,71^. Multi-heme cytochrome proteins were determined as ≥3 CXXCH, CXXXCH, or CXXCK motifs.

### Quinone extraction

Quinones were extracted from electrode biofilm from BES2 at the end of batch 5 using a modified Bligh & Dyer protocol^72^, dried under a flow of N_2_, and reconstituted in methanol for analysis. Analysis of quinones was performed using reversed-phase ultra-high performance liquid chromatography (UHPLC) coupled to mass spectrometry (MS) on a Vanquish Horizon UHPLC system equipped with a Waters Acquity BEH C18 column (150 × 2.1 mm, 1.7 μm particle size) coupled to a Q Exactive Plus Orbitrap high-resolution MS (Thermo Fisher Scientific, Bremen, Germany) equipped with a heated electrospray ionization source (HESI) operating in positive ionization mode according to the method of Wörmer et al.^73^. The injection volume was 10 µl, and quinones were eluted at a constant flow rate of 0.4 ml min^−1^ using linear gradients of methanol-water (85:15, vol/vol; eluent A) to methanol-isopropanol (50:50, vol/vol; eluent B), both with 0.04% formic acid and 0.1% NH_3_. The initial condition was 100% A held for 2 min, followed by a gradient to 15% B in 0.1 min and a gradient to 85% B in 18 min. The column was then washed with 100% B for 8 min. The column temperature was 65 °C. HESI and MS settings were optimized during infusion of a mixture of lipids including UQ10:10 and MK7:7 standards (Sigma Aldrich, St Louis, MO, USA). HESI settings were: capillary temperature, 250 °C; sheath gas (N_2_) pressure, 30 arbitrary units (AU); auxiliary gas (N_2_) pressure, 10 AU; spray voltage, 3.5 kV; probe heater temperature, 350 °C; S-lens 75 V. Quinones were detected by scanning from m/z 150 to 2000, followed by data-dependent MS2 (isolation window 1.2 m/z) using resolving powers of 140,000 and 17,500 (FWHM at m/z 200) in full-scan MS and MS2, respectively. Optimal fragmentation of quinones was achieved using a stepped normalized collision energy (NCE) of 15, 25, and 50. A mass calibration was performed using the Thermo Scientific Pierce LTQ Velos ESI Positive Ion Calibration Solution prior to analysis. Quinones were identified by retention time, accurate molecular mass, and MS^2^ fragment spectra^74,75^. Quinones were quantified by comparing their peak area to dilution series of MK_7:7_ and UQ_10:10_ standards.

### Biofilm activity assays

Activity assays with “*Ca*. M. vercellensis” enriched biofilms from BESs were performed after batch 4 in 60 mL serum bottles with 20 mL anoxic BES medium. In the anaerobic hood (97% N_2_, 3% H_2_), approximately one-sixteenth of the electrode biofilm of two different BESs was combined per bottle. The bottles were stoppered, the headspace was flushed with N_2_/CO_2_ (9:1) for 5 min, and pressurized to 1.1 bar. Subsequently, 10 mL ^13^C-CH_4_ was added, and the bottles were incubated at 26 °C and 200 rpm. In biological duplicate nitrate (250 µM), ferrihydrite (100 µM) and a control without electron acceptor were incubated for 8–9 days.

Biofilm activity from BESs were compared to bioreactor activity using 120 mL serum bottle subcultures of 60 mL (0.13 g dry weight) bioreactor samples in biological triplicate. Bioreactor biomass was washed three times in BES medium and made up to a final volume of 40 mL. Subsequent handling was identical to the BES biofilm bottle incubations, except that 20 mL ^13^C-CH_4_ was added instead of 10 mL. Nitrate and nitrite concentrations were checked twice a day using colorimetric test strips (detection limit 10 mg L^−1^, MQuant, Merck, Darmstadt, Germany).

### Protein phylogenetic analysis

A local protein database of MAGs with clear taxonomic classification from the Genome Taxonomy Database (GTDB R220) was used to search for OmcZ homologs using blastp. OmcZ homolog proteins were extracted from the MAGs with the *e*-value cutoff of 1e-20 and manual curation. In combination with homologs from the NCBI Clustered NR database and published literature, 90 OmcZ homolog proteins were identified across bacteria, archaea, and Borg elements. To obtain the sequences of the nanowire-forming OmcZ, the signal peptides were removed after the cleavage sites predicted by SignalP v5^76^, and the self-inhibitor C-part was removed after the cleavage sites of the accessory serine protease, which were chosen at the corresponding positions to the cleavage sites in *G. sulfurreducens* OmcZ^Gs^ (after WFGN)^27^ and “*Ca*. M. vercellensis” OmcZ2^Mv^ (after GWAG) from the multisequence alignment. This alignment also provides the conserved regions in Fig. 5A. The unrooted protein phylogeny tree of nanowire-forming OmcZ in Fig. 6 was calculated using the maximum likelihood method with 1000 bootstrap replications in MEGA-X (https://megasoftware.net/) and plotted in iTOL^77^.

### Nanowire purification

The yields of nanowires were examined during different purification steps of electrode-grown “*Ca*. M. vercellensis” samples: (1) the electrode-grown biofilm, HEPES based buffer, pH 7.25; (2) the supernatant of (1) after 21300 g spin; (3) the pellet of (1) after 21300 g spin and resuspended in 50 mM Tris 50 mM NaCl pH 8.0 buffer; (4) the 10-fold concentrated fraction (2). Based on Fig. 3C, fraction (1) exhibited a single major cytochrome band. Thus, for the following studies, 0.5 ml of electrode-grown medium was concentrated to ~80 μl using Amicon Ultra-0.5 ml 100 kDa cutoff centrifugal filters. To preserve the native conformations during purification, we avoided the pH 10.5 ethanolamine buffer or the 12.5% ammonium sulfate overnight precipitation used in prior studies^26–28^.

### Sodium dodecyl sulfate–polyacrylamide gel electrophoresis (SDS-PAGE) and heme staining gels

To thoroughly denature proteins, the samples were added to 4x Laemmli Sample buffer (BioRad, Cat. No 1610747) with 2.5% reducing reagent β-mercaptoethanol, boiled in a hotplate for 12 min, and then run on 4-20% gradient SDS-PAGE gels (Bio-Rad, Cat. No 4568093) powered by PowerPac Basic (Bio-Rad). The electrophoresis parameters were set to 190 V for 40 min. The standard marker of protein molecular weight was PageRuler Prestained Protein ladder (Thermo Scientific, product No. #26616). The gel in Fig. 3c was stained for 1 h with GelCode Blue Safe Protein Stain (Thermo Scientific, ref. 1860957) and overnight destained in deionized water. The heme staining gel in Fig. 3e was soaked in a mixed solution of 15 mL methanol, 30 mg TMB (3,3,5,5-tetramethylbenzidine), and 35 mL 0.5 M sodium acetate, pH 5.0, under dark conditions. Heme-containing bands turned blue after incubation with 300 μL H_2_O_2_ for another 5 min^78^. No β-mercaptoethanol was used during heme staining to prevent interference with the color reaction.

### Protein mass spectrometry

Both samples from electrode-grown culture medium and carefully excised ~70 kDa SDS-PAGE gel bands were sent to the Keck Mass Spectrometry & Proteomics Resource at Yale University. Subsequent proteolytic digestion following reduction and alkylation were carried out, after which peptides were analyzed using a Waters Acquity M-class UPLC system coupled to a Thermo Scientific Q Exactive HF-X mass spectrometer. Peptides were separated over a 0–90 min gradient. Solvent A consisted of H₂O containing 0.1% formic acid, and solvent B consisted of acetonitrile in H₂O with 0.1% formic acid. The Orbitrap mass spectrometer was operated in data-dependent acquisition (DDA) mode, acquiring peptide signals from m/z 300–1700 at a resolution of 70,000 in full MS mode, with a maximum ion injection time of 45 ms and an automatic gain control (AGC) target of 3 × 10⁶. The top 20 precursor ions were selected for MS/MS analysis and fragmented using higher-energy collisional dissociation. MS/MS scans were acquired at a resolution of 17,500 with an AGC target of 1 × 10⁵, a maximum injection time of 100 ms, an isolation width of 1.7 m/z, and a NCE of 28. Singly charged ions and ions with charge states ≥7 were excluded from fragmentation. Peptide match was preferred, and dynamic exclusion was set to 20 s.

Mass spectrometric raw data were analyzed against a protein database derived from genome sequencing using PEAKS Studio X (Bioinformatics Solutions Inc., Waterloo, Canada). Database searches were performed with a precursor mass tolerance of 20 ppm and a fragment ion mass tolerance of 0.02 Da, allowing up to 3 missed cleavages. Carbamidomethylation was included as a fixed modification, and methionine oxidation, as well as asparagine and glutamine deamidation, were included as variable modifications. Peptide-spectrum matches were filtered to a 1% false discovery rate (FDR), and protein identifications supported by at least two unique peptides were considered significant. Results from PEAKS were exported to proteins.csv, listing the identified proteins.

### Negative staining transmission electron microscopy (TEM)

The plasma discharger (HARRICK PLASMA, PDC-001-HP) cleaned the CF400-CU grids (Electron Microscopy Sciences) at low power. The purified nanowires were drop-cast onto the cleaned grids and adsorbed for 10 min. Then, after removing the excessive liquid, the grids were carbon-side facing down on 50 μL droplets of 1% phosphotungstic acid. Finally, the grids were allowed to air dry after the excess stain was removed. TEM images were recorded using a JEM-1400 (JEOL) microscope operated at 80 kV on the Yale West Campus.

### Cryo-EM sample preparation, data collection and image processing

The purified nanowires (3 μL) were added gently to plasma-discharged cryo-EM grids (C-Flat, CF-2/1-3Cu-50, Cat #CF312-50). The cryo-EM specimen was prepared in the Vitrobot Mark IV System (Thermo Scientific), with the temperature set to 22 °C and the humidity to 100%. The samples on grids were backblotted onto a single filter paper and plunge-frozen in liquid ethane. Micrographs were collected by a Glacios cryo-TEM (Thermo Scientific) operating at 200 kV with a K3 camera (Yale Science Hill cryo-EM facility). The movies were acquired using super-resolution imaging mode with a physical pixel size of 0.868 Å and a total dose of 40 e^-^/Å^2^. The cryo-EM data analyses were conducted in cryoSPARC^79^. The motion corrections and contrast transfer function estimations were applied first, then the particles were auto-picked with “Filament tracer” and cleaned by multiple rounds of 2D classification. 210,625 good particles were selected for the construction of the density map. The helical symmetry was calculated by Spring^80^ on negative-stain TEM images and fine-tuned by the “Symmetry search utility” in cryoSPARC^79^. Helical symmetry parameters, including helical rise and twist, were determined using as a helical rise of 58.0 Å and a twist of 159.5°. Map FSC was used to estimate the reconstruction’s resolution. An overview of the workflow is presented in Supplementary Fig. 13.

### Model building of OmcZ2^Mv^ nanowires

The amino acid sequence of OmcZ2^Mv^ was retrieved from the closed genome sequence from this work and used as input for AlphaFold3^81^ to generate predicted structural models. The predicted models were fitted into the cryo-EM map using ChimeraX^82^, followed by real-space refinement in PHENIX^83^ and Coot^84^. The final protomer models exhibited excellent stereochemistry, as evaluated by PHENIX. To better refine heme-interacting areas at this resolution, bond/angle restraints for the heme molecule itself (His-Fe) and Cys-heme thioester bonds were restricted based on the geometries obtained in the reported structure of OmcZ nanowires from *Geobacter sulfurreducens* (PDB 7LQ5). Phenix reported a map-to-model correlation coefficient of 0.85, which served as robust cross-validation metric for evaluating map quality and the accuracy of helical symmetry parameters. The helical OmcZ2^Mv^ filament was modeled in Phenix by fitting 3 subunits of OmcZ2^Mv^ filament monomers into the helical cryo-EM density map. UCSF ChimeraX^82^ was utilized for surface rendering of cryo-EM density maps and for segmentation in all visual presentations of structures. All the structures were validated in PHENIX^83^ using the Comprehensive validation tool, and the map quality was assessed using the Mtriage tool in PHENIX^83^, as summarized in Supplementary Table 6. Structural models of full-length OmcZ^Gs^ and OmcZ2^Mv^ were predicted by AlphaFold3^81^. ChimeraX^82^ was used for all visual exhibitions of structures.

### Reporting summary

Further information on research design is available in the Nature Portfolio Reporting Summary linked to this article.

## Supplementary information

Supplementary Information - NEW

Description of Additional Supplementary Files

Supplementary Data 1

Supplementary Data 2

Reporting Summary

Transparent Peer Review file

## Source data

Source Data

## Data Availability

Sequencing data and MAGs have been deposited in the European Nucleotide Archive (ENA) under bioproject PRJEB98224. The chromosome of *Ca*. M. vercellensis is deposited within the bioproject under accession “GCA_988232875.1” whereas the associated plasmid has been published previously under bioproject PRJNA850006 as biosample SAMN31367896 (https://ncbi.nlm.nih.gov/nuccore/CP113316.1). Structural data has been deposited in the RCSB Protein Data Bank under accession 10RP. Proteomic data is available on the PRIDE database under accession PXD072602. Source data are provided with this paper.
