# Supplementary material for: Electrode-associated growth reveals structure of OmcZ-type cytochrome nanowires in anaerobic methanotrophic archaea
Source: Nat Commun. 2026 Nov 9;17:11117. doi: 10.1038/s41467-026-78976-x (PMC13748539; doi:10.1038/s41467-026-78976-x)
Supplement: Supplementary file 1 [file 41467_2026_78976_MOESM1_ESM.pdf]

Supplementary data to

Electrode-associated growth reveals structure of OmcZ-type cytochrome nanowires in anaerobic methanotrophic archaea

Martijn Wissink[1,8], Cong Shen[2,3,8], Jiaqi Wang[2,4], Reinier A. Egas[1], Phoenix Smid[1], Felix J. Elling[5], Kevin W. Becker[6], Martin Pabst[7], Mike S. M. Jetten[1], Jun Liu[3,4], Nikhil S. Malvankar[2,3]*, Cornelia U. Welte[1]*

[1] Department of Microbiology, Radboud Institute for Biological and Environmental Sciences, Radboud University, 6525AJ Nijmegen, the Netherlands
[2] Microbial Sciences Institute, Yale University, West Haven, CT 06516, USA
[3] Department of Molecular Biophysics & Biochemistry, Yale University, New Haven, CT 06511, USA
[4] Department of Microbial Pathogenesis, Yale University, New Haven, CT 06511, USA
[5] Leibniz-Laboratory for Radiometric Dating and Isotope Research, Christian-Albrechts-University of Kiel, 24118 Kiel, Germany
[6] Faculty of Biology/Chemistry, University of Bremen, 28359 Bremen, Germany
[7] Department of Biotechnology, Delft University of Technology, 2629 HZ Delft, The Netherlands
[8] These authors contributed equally
*Correspondence: nikhil.malvankar@yale.edu (N.S.M.), c.welte@science.ru.nl (C.U.W.)

Content: This document contains Supplementary Figures 1-13, and Supplementary Tables 1-6.

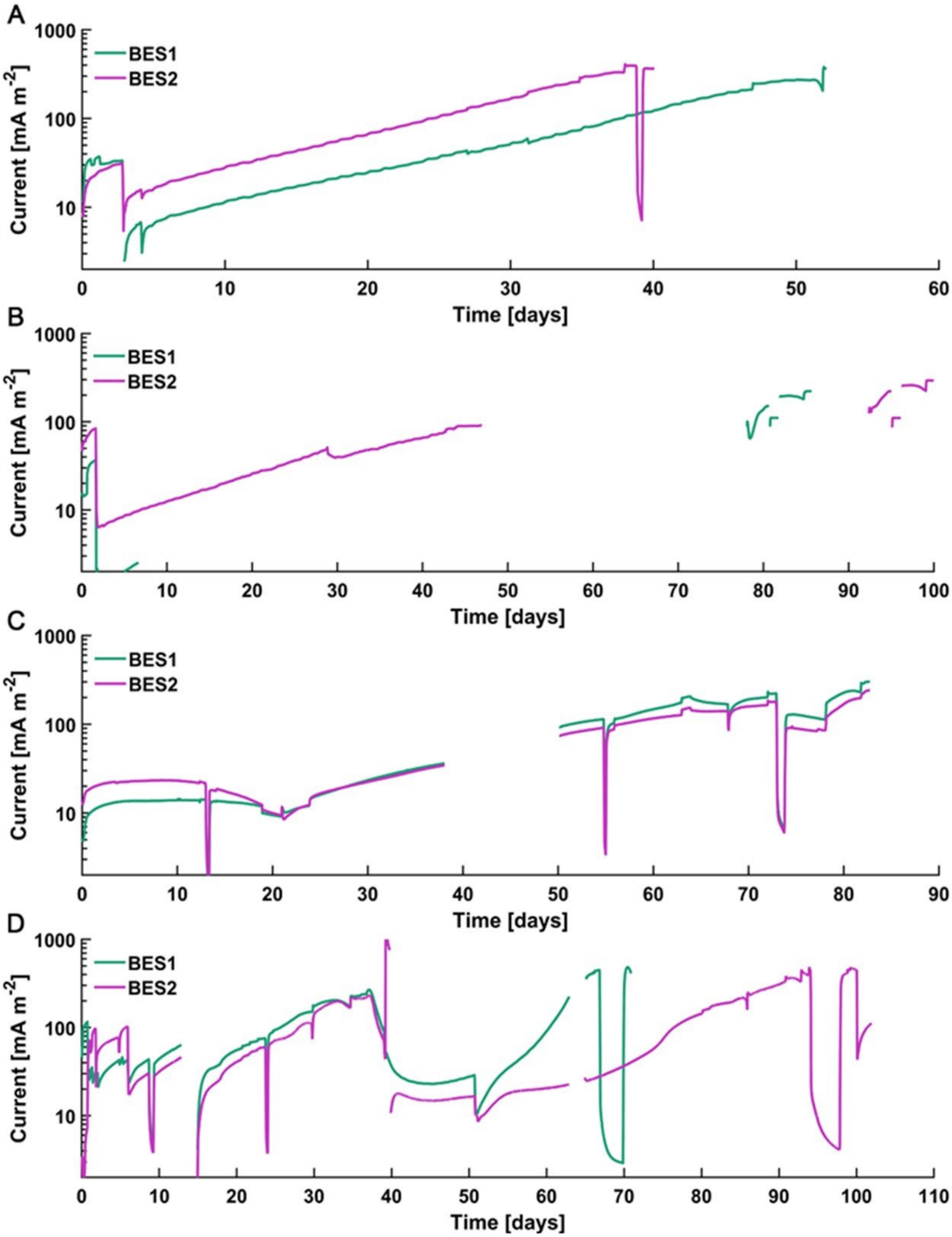

Supplementary Figure 1. Transfer of electrode biofilm results in renewed exponential current increase.

(A) Clean duplicate BESs were inoculated with electrode pieces containing biofilm from the first incubation (Figure 1 A & B). Compared to the first batch the second batch showed a reduced lag phase and could reach similar current densities as the first batch within 30 days. (n=2 biological replicates)

(B–D) After maximum current output was reached, electrode biofilm pieces were again transferred to fresh BESs. Occasional data gaps reflect technical issues during data recording. Source data and BES logs can be found in Source Data. (n=2 biological replicates)

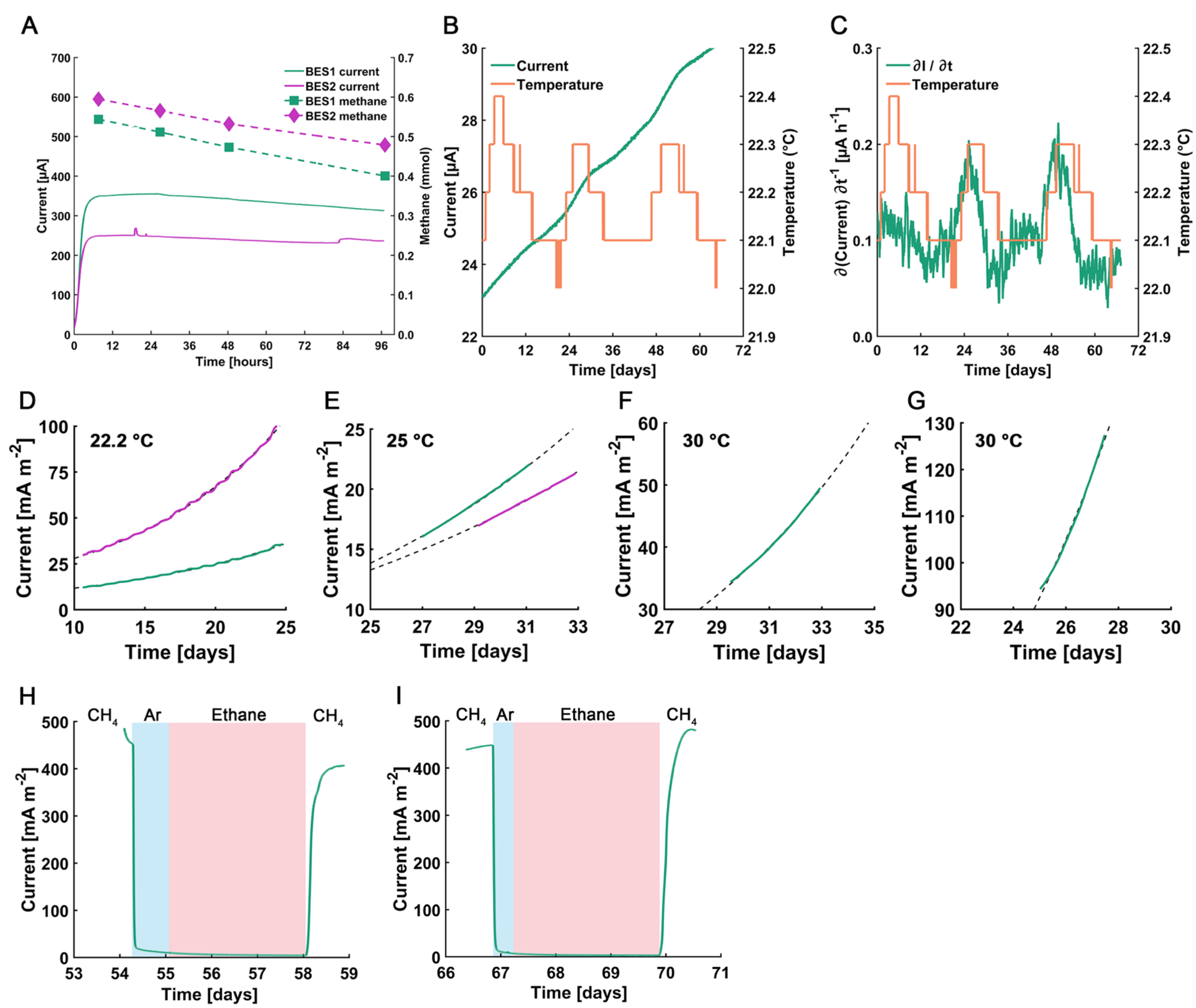

Supplementary Figure 2. Physiological characterization of '*Ca.* M. vercellensis' growth on electrodes.

(A) Coulombic efficiency of methane respiration on electrodes. In duplicate closed BESs, current and methane concentrations were measured to calculate coulombic efficiency. (n=2 biological replicates)

(B-C) Current is temperature dependent and follows a diurnal pattern. (B) Current and temperature as a function of time. (V) Rate of current change (∂I / ∂t) plotted alongside temperature.

(D-G) Exponential current increase as a proxy for microbial growth: additional data to Figure 3. Exponential current increase was observed at 22.2 °C (D), 25 °C (E), and 30 °C (F, G). Exponential trendlines (black dashed lines) were fitted to the data. Data from BES1 are shown in green and BES2 in pink.

(H) Ethane does not support current generation. Methane was replaced by argon (blue bar), followed by addition of 20 mL ethane (pink bar) with current shown in green. Methane flushing was resumed after two days. (n=2 biological replicates, the second replicate shown in (I)).

(I) Biological duplicate of Figure S2H. (n=2 biological replicates)

Source data are provided as a Source Data file.

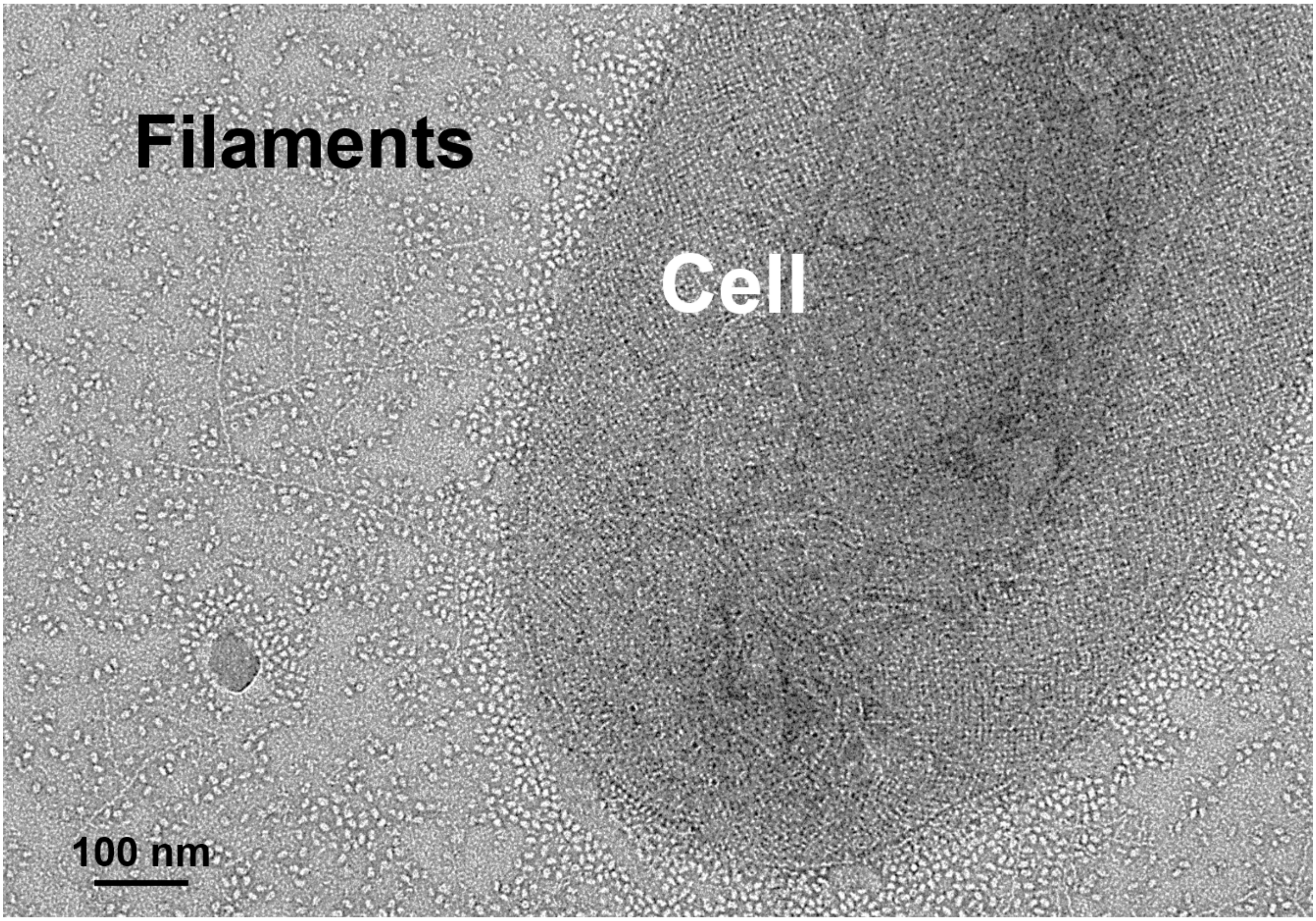

Supplementary Figure 3. Negative-stain TEM image of '*Ca.* M. vercellensis' grown on electrodes. S-layer containing cells presumably represent '*Ca*. M. vercellensis' with nanowire-like appendages associated to the cell surface. Imaging was performed three times (n=3).

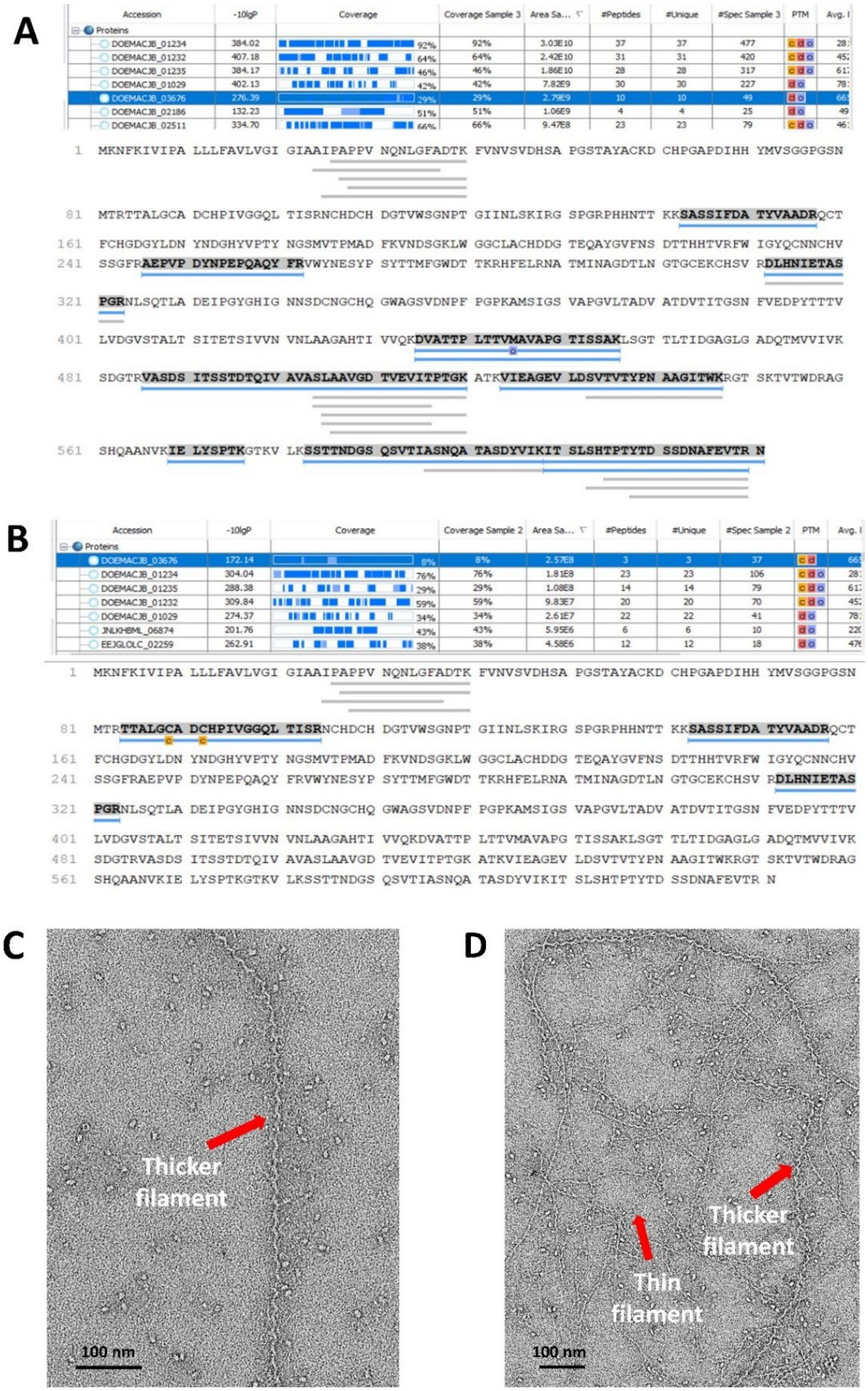

Supplementary Figure 4. Mass spectrometry identification of OmcZ2^{Mv} and another thicker filament of electrode-grown '*Ca*. M. vercellensis', related to Figure 3.

(A-B) OmcZ2^{Mv} (DOEMACJB_03676) is one of the most abundant proteins in (A) the culture medium where both the nanowire-forming part and the cleaved C-terminal part were present; (B) the 70 kDa protein band where mainly the nanowire-forming part was present.

(C-D) Another type of thick sinusoidal filaments was observed in negative stain TEM imaging. Imaging was performed three times (n=3).

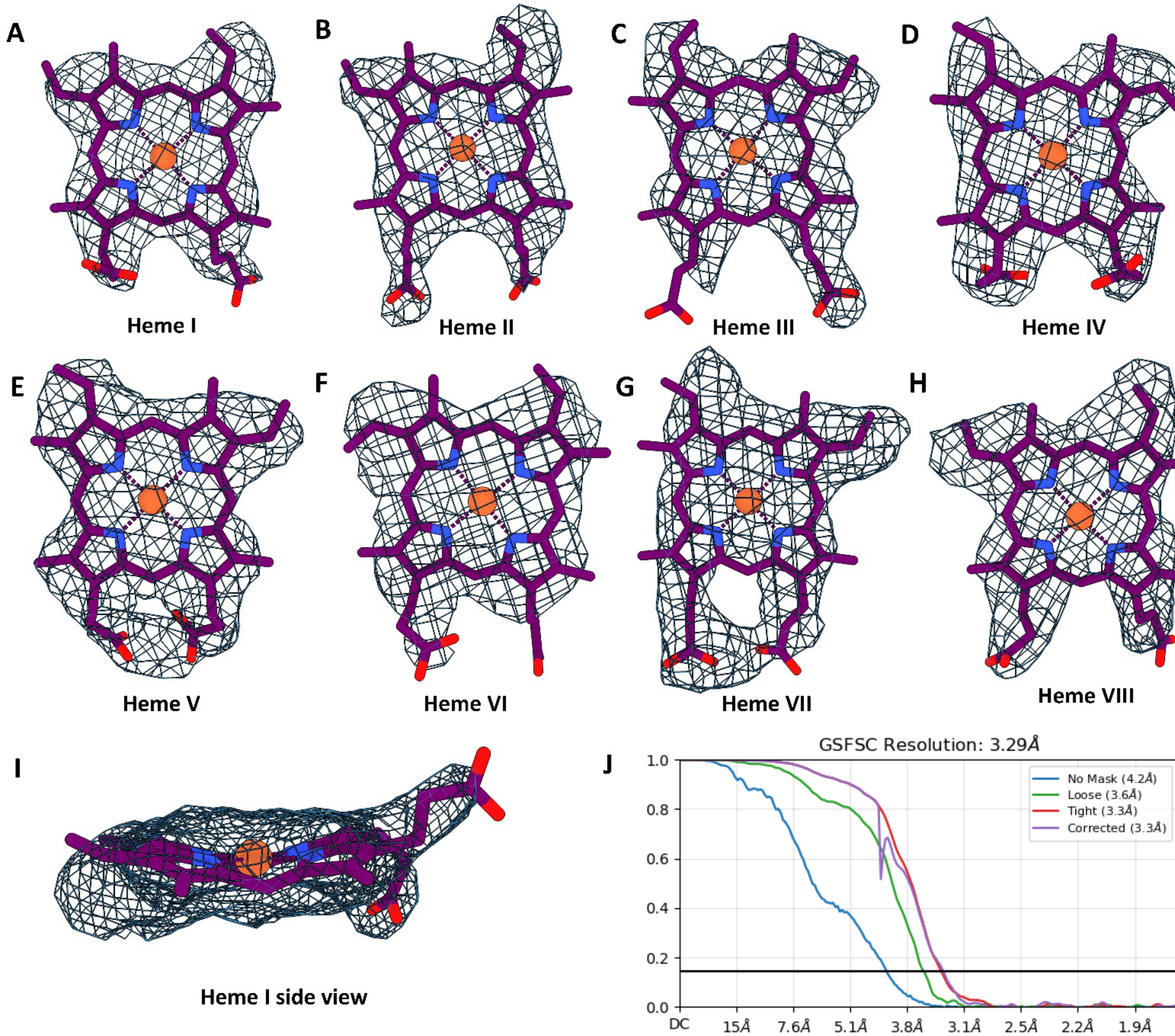

Supplementary Figure 5. Illustration of cryo-EM map quality.

(A-H) The top view of electron density fitting of each heme co-factor.

(I) The side view of electron density fitting of Heme I.

(J) Map:map Fourier shell correlation (FSC) curves with different masks exhibit the structural resolution at 3.3 Å.

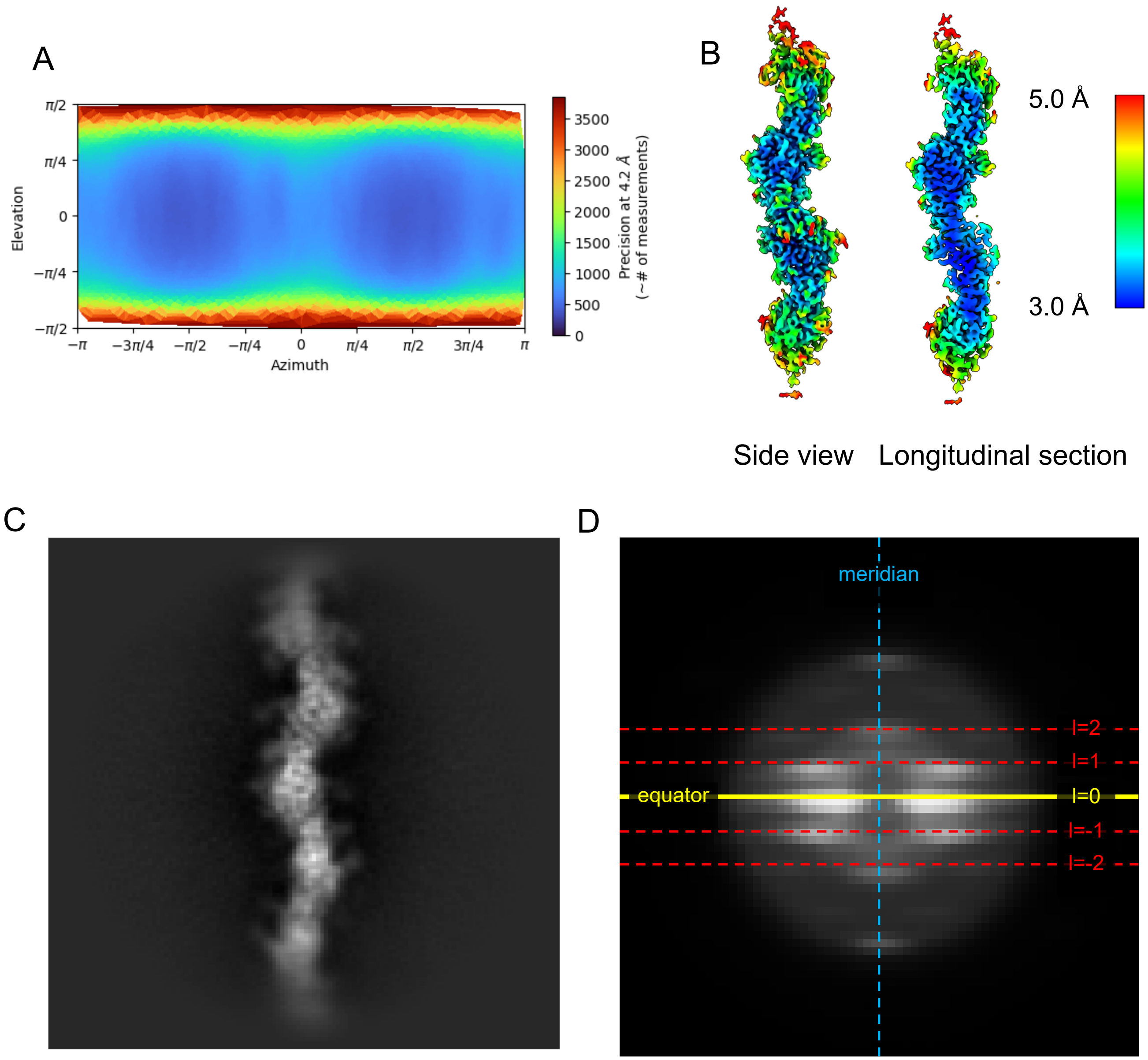

Supplementary Figure 6. Additional cryo-EM data quality indicators.

(A) Particle orientation distribution.

(B) Local resolution analysis shown as a resolution-colored surface.

(C, D) Power spectrum from selected 2D class average using the Average Power Spectra job in cryoSPARC (v4.7.1)

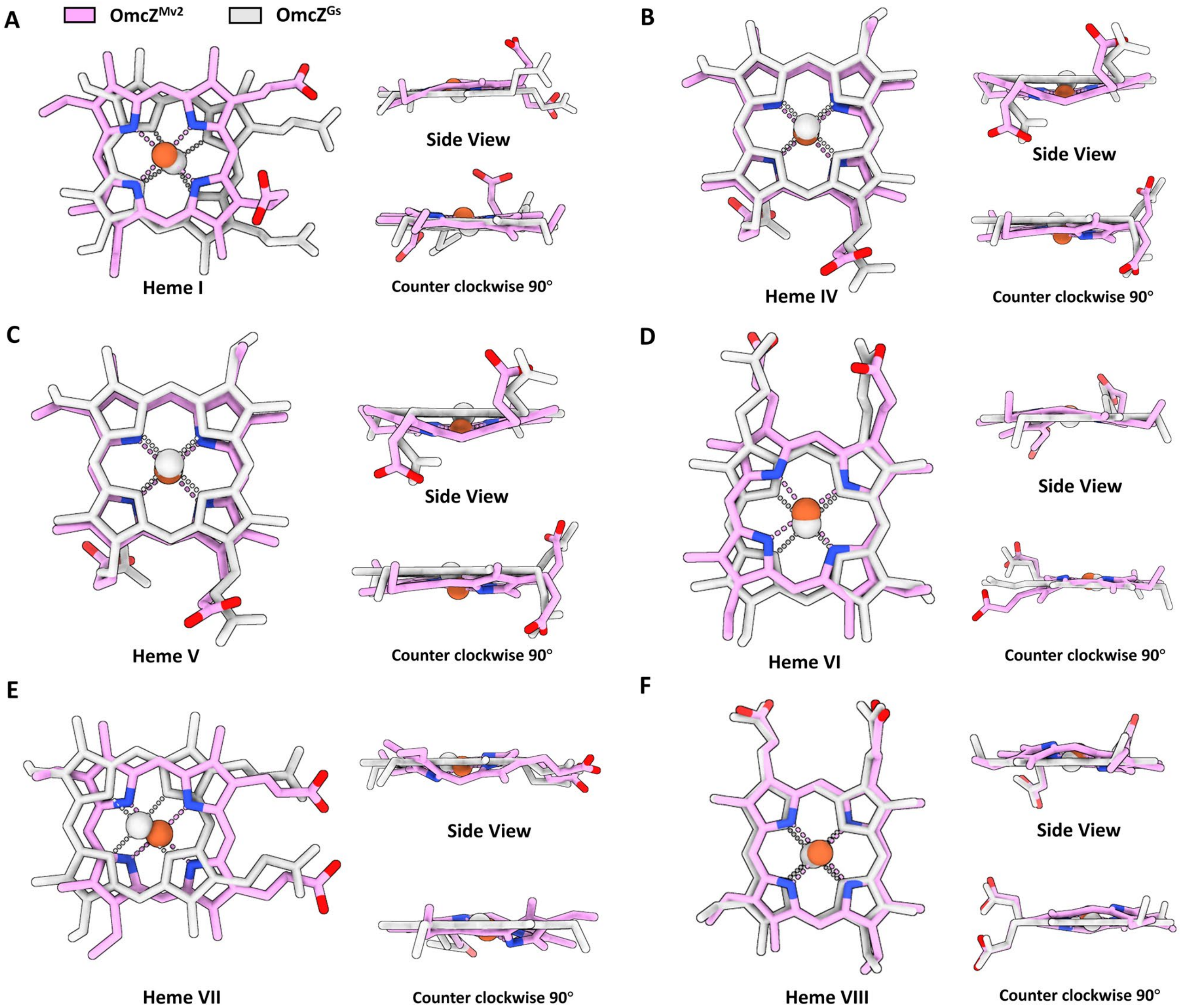

Supplementary Figure 7. Alignments of individual hemes other than Heme II & III between OmcZ2^{Mv} (pink) and OmcZ^{Gs} (light grey), related to Figure 3.

Hemes in OmcZ2^{Mv} exhibit non-planar deformation to various extents. (A) Heme I; (B) Heme IV; (C) Heme V, (D) Heme VI, (E) Heme VII, (F) Heme VIII. The structure of OmcZ^{Gs} was obtained from PDB: 7LQ5.

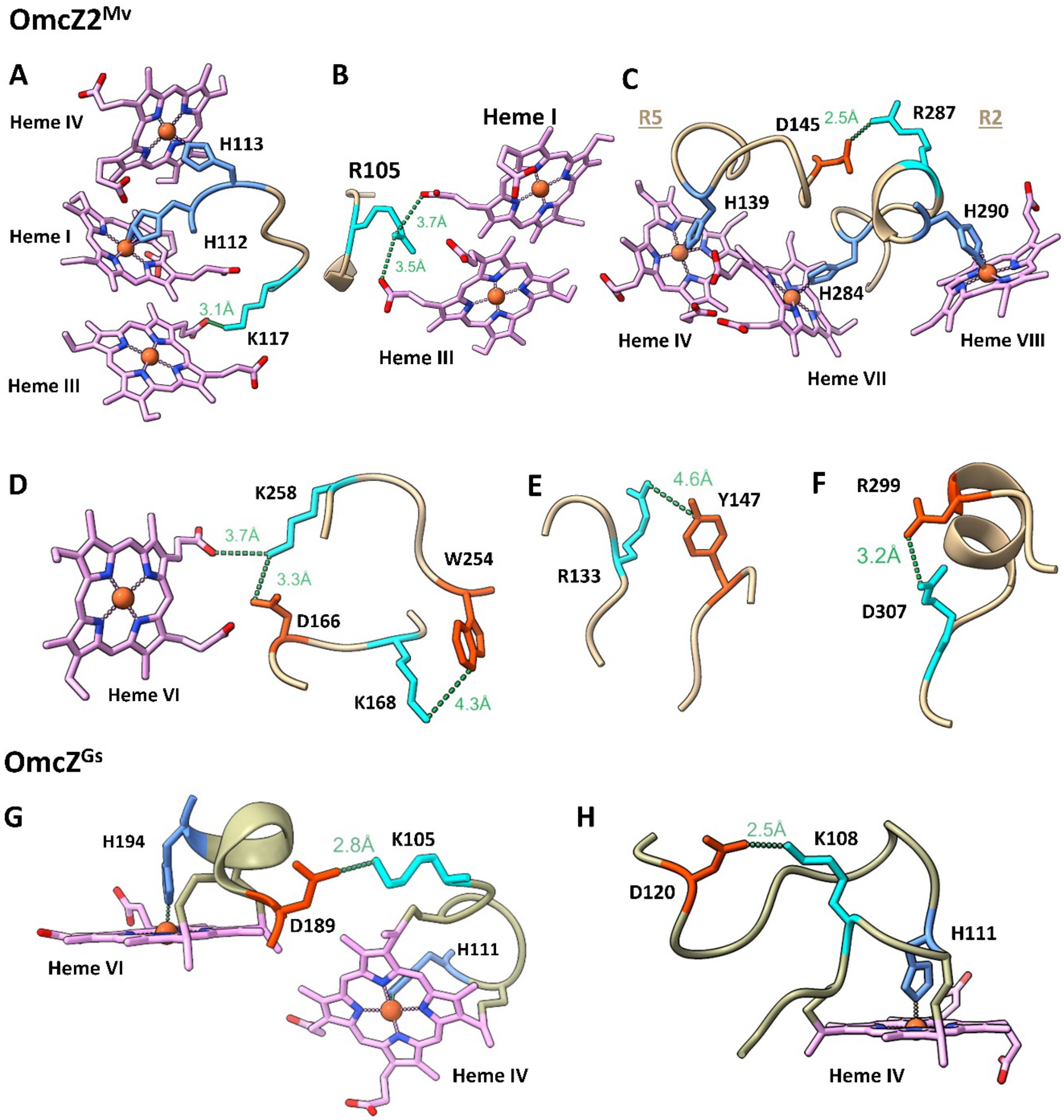

Supplementary Figure 8. Other unique interactions maintaining structural integrity of OmcZ homologues, related to Figure 4.

(A-C) In OmcZ2^{Mv}, hemes are connected via salt bridges: (A) Heme III and Heme I & IV are connected via a salt bridge between the propionate of Heme III and K117; (B) Heme I & III are connected via the salt bridge between their propionates and R105; (C) Heme IV and Heme VII & VIII are connected via a salt bridge between D145 and R287.

(D) In OmcZ2^{Mv}, the Heme VI-VII expansion shown at the top loop (254-258) is anchored by the cation-π interaction between W254 and K168, and the salt bridge between K258 and D166. K258 from the expansion region also forms a salt bridge with Heme VI.

(E-F) In OmcZ2^{Mv}, some loops are stabilized by: (E) the cation-π interaction between R133 and Y147, and (F) the salt bridge between D307 and R299.

(G-H) In OmcZ^{Gs}, Heme IV is connected to (G) Heme VI via a salt bridge between K105 and D189, and (H) another loop via a salt bridge between K108 and D120.

A **>OmcZ2^{Mv} 11 NXS/T sites**

IPAPPVNQNLGFADTKFVNVSVDHSAPGSTAYACKDCHPGAPDIHHYMVSGGPGSNMTRTTALGCA
DCHPIVGGQLTISRNCHDCHDGTVWSGNPTGIINLSKIRGSPGRPHHNTTKKSASSIFDATYVAADRQC
TFCHGDGYLDNYNDGHYVPTYNGSMVTPMADFKVNDSGKLWGGCLACHDDGTEQAYGVFNSDTT
HHTVRFWIGYQCNNCHVSSGFRAEPVPDYNPEPQAQYFRVWYNESYPSYTTMFGWDTTKRHFELRN
ATMINAGDTLNGTGCEKCHSVRDLHNIETASPGRNLSQTLADEIPGYGHIGNNSDCNGCHQGWAG

B **>OmcZ^{Gs} 3 NXS/T sites**

AVPPPPVNQFLGIYDTKFPNLTKADCLECHVSDTVLVQQHHALINTVTPPASCINTSGTVPPTLATGCHV
MVPDGSGGFTFQDFRNCFNCHTQTPHHTSPAAVAKDCKYCHGNFIDNPLDGHYIPTYSASSVTPMPS
GRSVTATDGNVVIVQGCEACHQAAPNAIDPKTNTVRPIFSNQDTHHGTGITDCNLCHNTSSNVPIRQC
EVCHGVNSLHNIQKDSPNAANLGTVKPGLEDLGWGHIGNNWDCQQGCHWSWFGN

C **>Amber_301, 16 NXT/S sites**

SVFVPPPPPVPQSIGLYDTNIANLVTNATDQSACRKCHQTTGTNVSLVNNGTYNNTVGGVPTRHHSLVE
RAITNPLTGQPFGCEDCHPANQGFGNGILLDRSCVNCHNGSSFYADATQGANVGNFSRPHHVITNYTS
VVPNFGNPAADRQCNVCHGSFVANYNDGHYRPTYATTFDITPFATFKATSNVTANAETPPDADDNKT
WGGCFSCHEDAPAVGGPIGSNHDNHHLSILGNSSANSSAFQKLATPFDLSYAPGGRACFVCHVVATTGS
PLRVNITDGGTGLTVRAMEVRNSTLEFGSILGIVSDPEPGTTNISINGTGCEKCHDVKSLHNINAPATYTR
NGPQGVGHIQNNTDCEGCHLSAVPLDTN

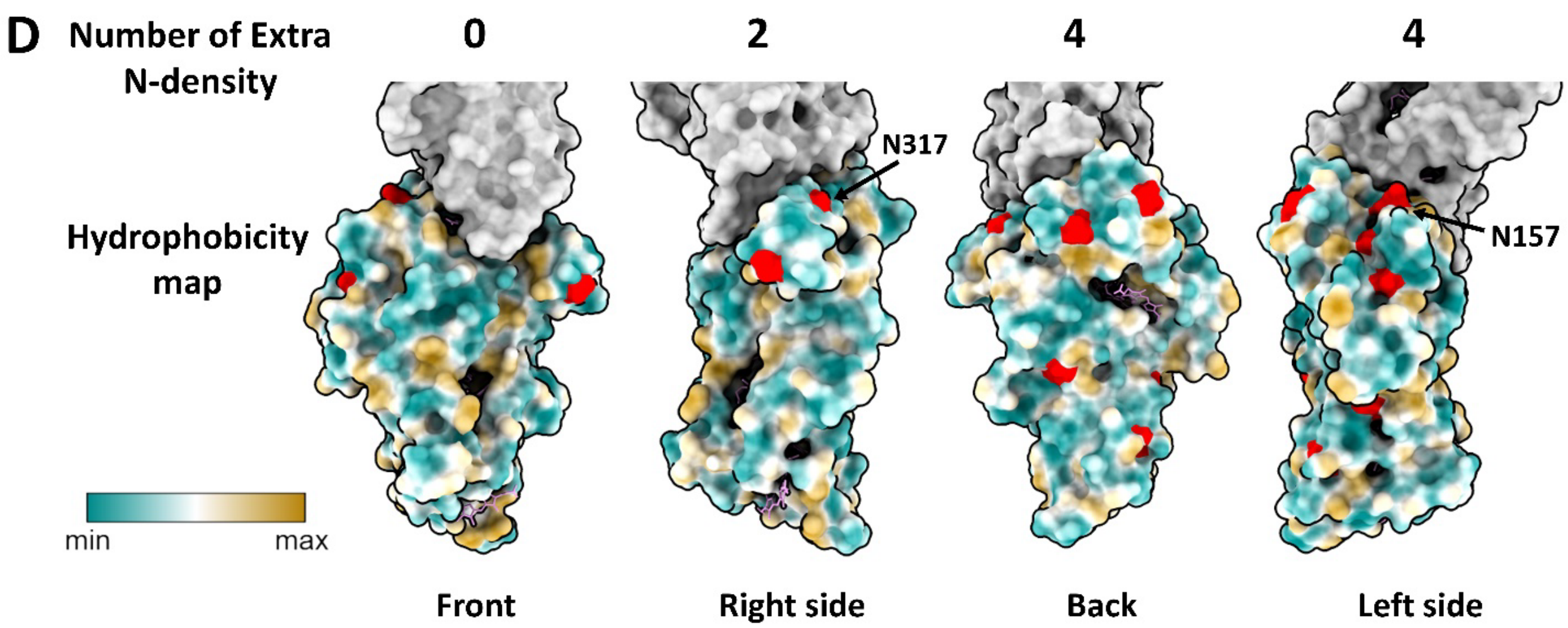

Supplementary Figure 9. The high percentage of N-glycosylation motifs NXS/T (X ≠ P) among the group I-IV, related to Figure 4.

(A-C) Protein sequences of the nanowire-forming parts of (A) OmcZ2^{Mv} (archaeal), (B) OmcZ^{Gs} (bacterial) and (C) Amber_301 (Borg). NXS/T motifs with observed extra densities are underlined and highlighted in red. NXS/T motifs without structural verification are underlined and highlighted in green. Heme binding motifs CXXCH are underlined and highlighted in blue.

(D) The 10 asparagine residues with extra density (red) are unevenly distributed near the hydrophobic regions on OmcZ^{Mv2} surface. Dark cyan color represents hydrophilic regions, and dark golden color represents hydrophobic regions. The subunit above is marked in light grey.

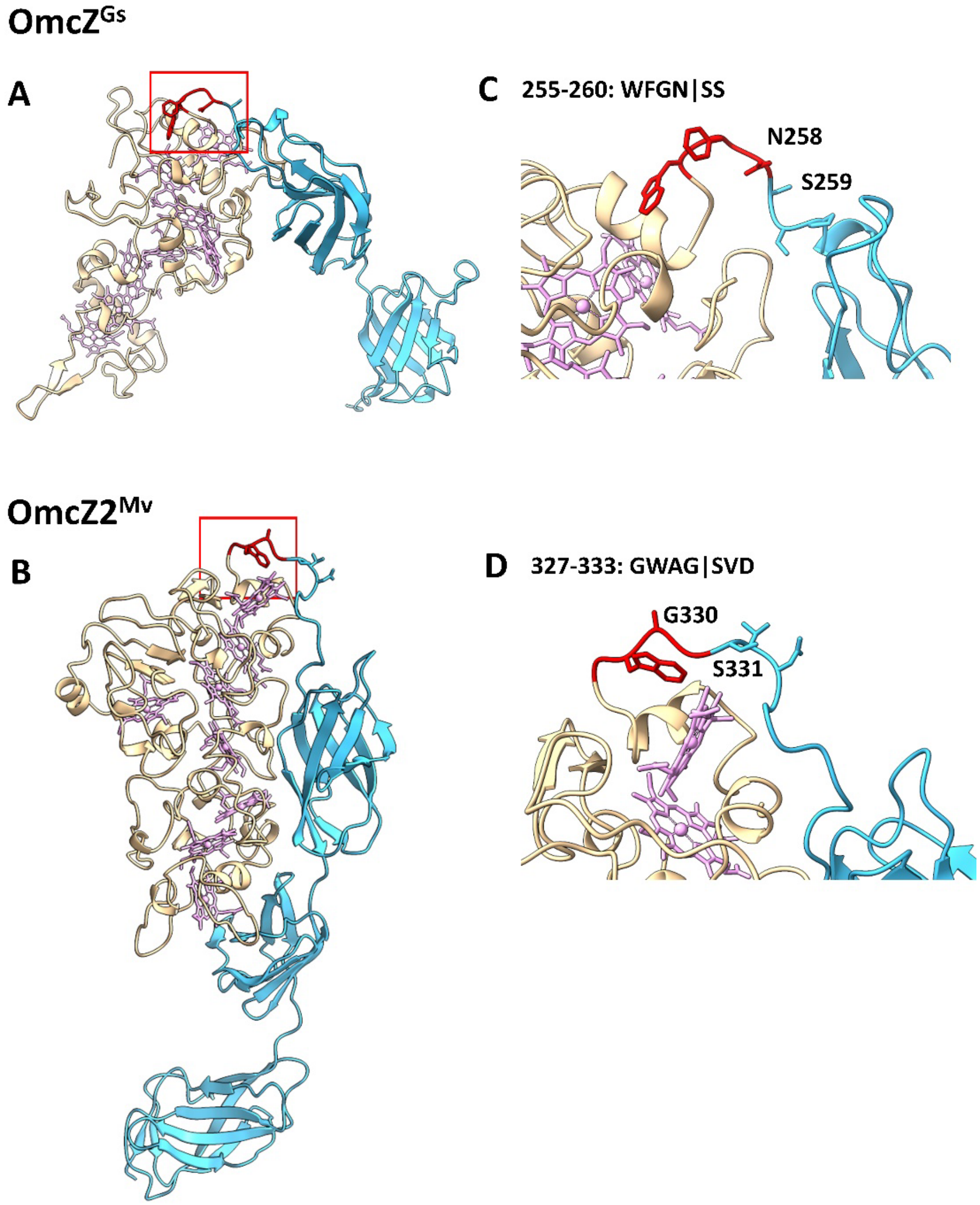

Supplementary Figure 10. Cleavage sites of the C-terminal part are conserved between and OmcZ^{Gs} and OmcZ^{Mv2}, related to Figure 5.

(A-B) The models of full-length OmcZ^{Gs} (A) and OmcZ2^{Mv} (B) are predicted by AlphaFold3. The nanowire-forming N-terminal parts are highlighted in beige with the heme chains highlighted in pink. The cleaved C-terminal parts are highlighted in blue. The residues before the cleavage site are highlighted in red.

(C-D) The close-up views of the cleavage sites of OmcZ^{Gs} (C) and OmcZ2^{Mv} (D). Residues before the cleavage sites, WFGN (255-258) in OmcZ^{Gs} (C) and GWAG (327-330) in OmcZ2^{Mv} (D) are highlighted in red. S259 in OmcZ^{Gs} (C) and S331 in OmcZ^{Mv2} (D) are behind the cleavage sites.

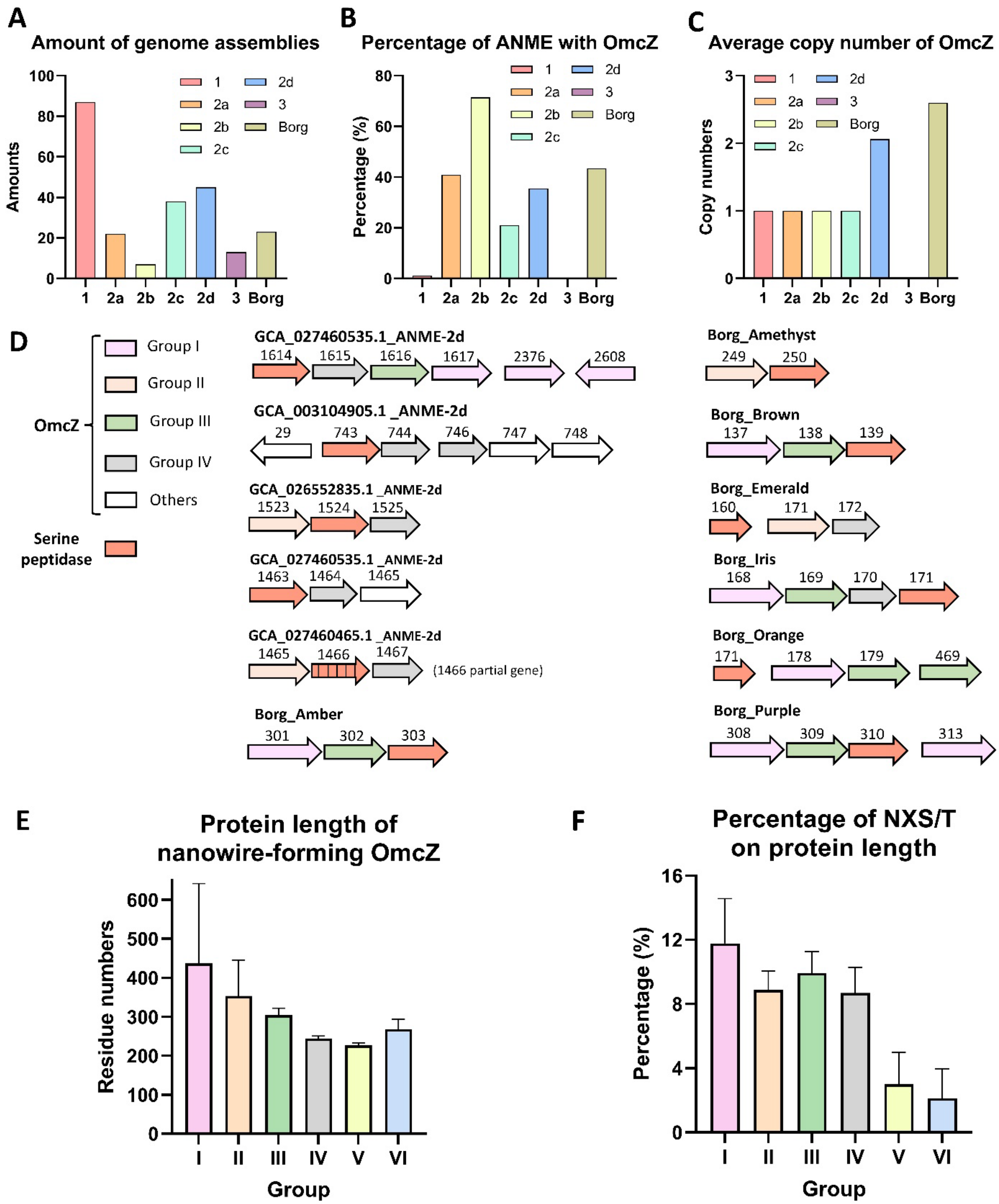

Supplementary Figure 11. The distribution of OmcZ homologues and their cluster arrangements across ANME archaea and the related Borg elements, related to Figure 6. (A) The amount of assembled ANME genomes across all lineages (1, 2a-d, 3) and Borg elements analyzed in this study. (B) The percentage of ANME genomes and Borg elements in (A) with OmcZ homologues. (C) The average copy numbers of OmcZ homologues in ANME genomes and Borg elements which have OmcZ homologues. (D) Other gene arrangements of OmcZ clusters in the ANME-2d lineage and Borg elements, apart from what are listed in Figure 6. Borg elements are labeled with color names. OmcZ homologues from various groups which are marked with different colors are encoded in several ANME-2d archaeal genomes and Borg elements. The OmcZ-associated serine peptidases are marked in red arrows. The number on top of each arrow is its gene ID. (E-F) The comparison of (E) the protein length and (F) NXS/T percentage of nanowire-forming OmcZ homologues across all 6 groups. Error bars indicate standard deviation

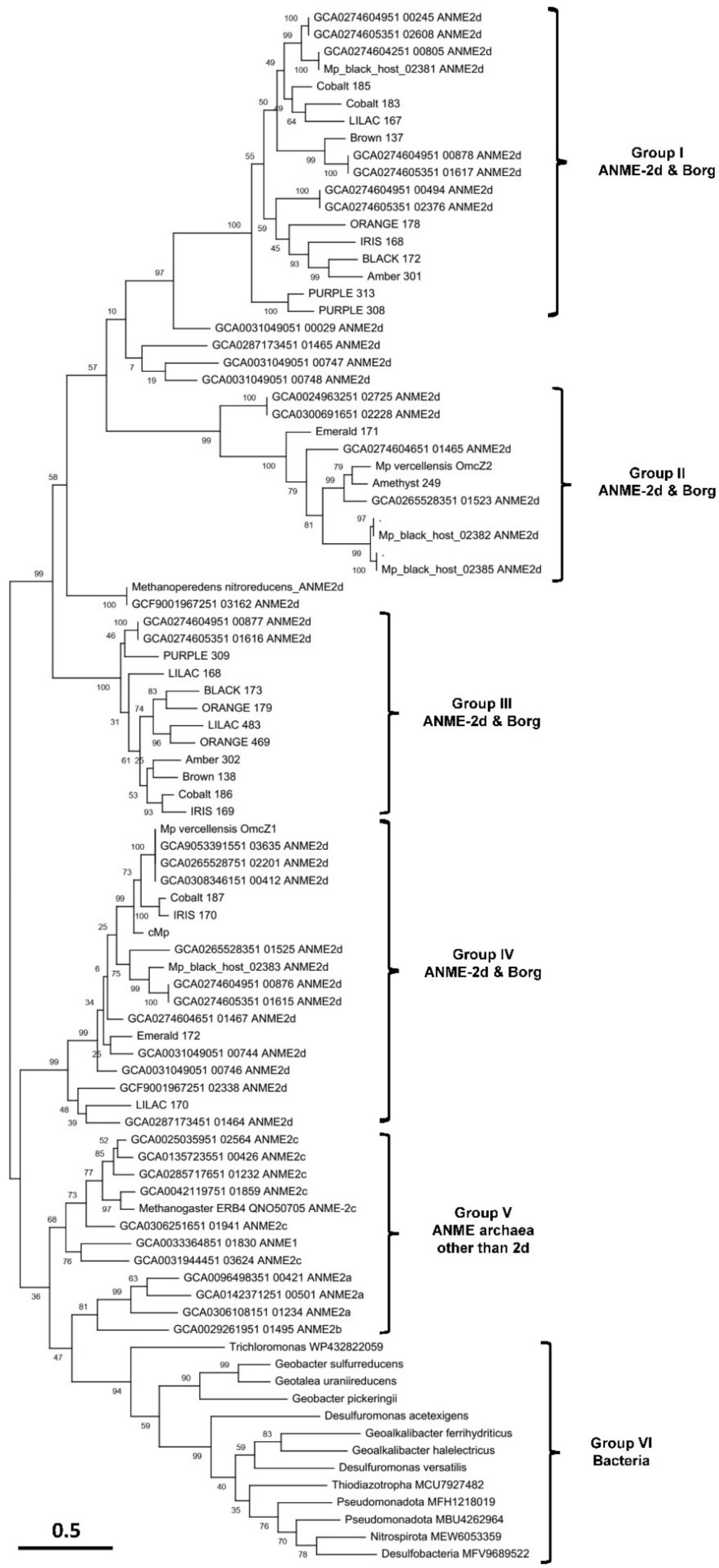

Supplementary Figure 12. The unrooted protein phylogeny tree of nanowire-forming N-terminal part from OmcZ homologues, related to Figure 6. The OmcZ homologues are chosen from bacteria, archaea and Borg elements. The species names are listed next to nodes. The ANME lineages are listed behind the archaeal names. Borg elements are named based on colors. Bootstrap values are listed on each branch points. ANME genomes were retrieved from the GTDB R220.

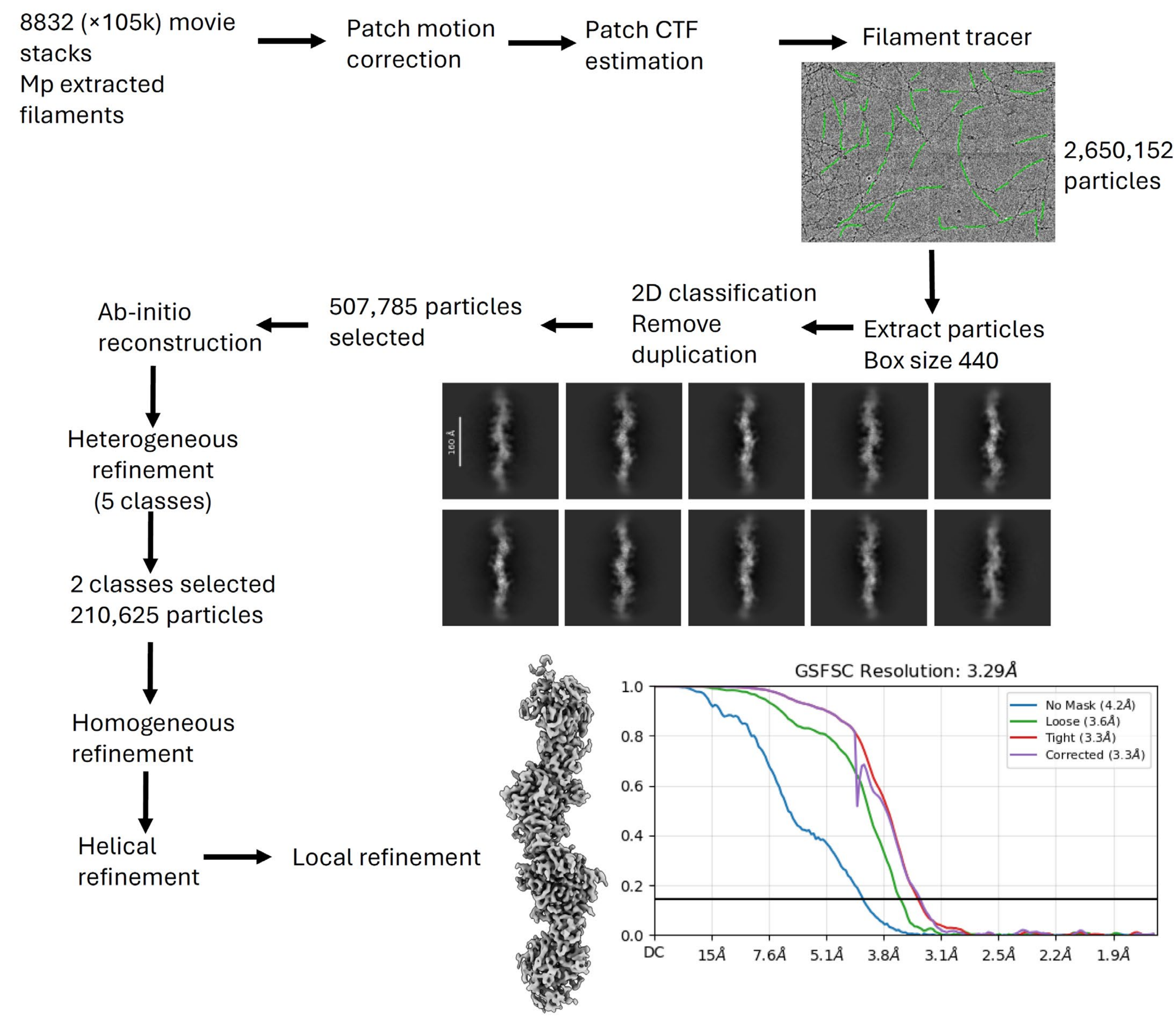

Supplementary Figure 13: Cryo-EM workflow.

Supplementary Tables

Supplementary Table 1. Genome information of '*Ca.* M. vercellensis' and its extra chromosomal element HMp_v2.

	**'*Ca.* M. vercellensis'**	**Extra chromosomal element HMp_v2**
**Completeness**	98.8%	4.17%
**Contamination**	5.18%	0%
**Genome size**	3.41 Mbp	0.156 Mbp
**GC content**	41%	39%
**Coding sequences**	3741	157

Supplementary Table 2. Quinones detected in the electrode-associated microbial community and their abundance relative to total quinones. Some quinones occur as isomers, likely due to cis/trans isomerism[1], and are denoted as (a) for the early-eluting peak and (b) for the late-eluting peak.

**Quinone**	***m/z* [M+H]$^+$**	**Abundance (%)**
$UQ_{10:10}$ (a)	863.6912	0.04
$UQ_{10:10}$ (b)	863.6912	4.22
$UQ_{9:9}$ (a)	795.6286	0.04
$UQ_{9:9}$ (b)	795.6286	1.22
$UQ_{8:8}$ (a)	727.5660	0.01
$UQ_{8:8}$ (b)	727.5660	1.05
$MK_{10:10}$	853.6857	0.75
$MK_{9:9}$	785.6231	20.35
$MK_{8:7}$	719.5762	3.57
$MK_{8:8}$	717.5605	67.03
$MK_{8:9}$ (a)	715.5449	0.23
$MK_{8:9}$ (b)	715.5449	0.08
$MK_{7:7}$	649.4979	1.40
$MK_{6:6}$	581.4353	0.01

Supplementary Table 3. Comparison of basic structural information between OmcZ^{Gs} and OmcZ2^{Mv} related to Figure 3. Data on OmcZ^{Gs} was retrieved from PDB 7LQ5.

	OmcZ^{Gs}	OmcZ2^{Mv}
Full length	473	631
Filament-forming length	258	330
Heme binding motifs	8	8
Helical rise	57.4	58.0
Helical twisting angle	-159.1°	-159.5°
α-helix	15.9%	16.4%
β-sheet	2.3%	1.8%
Coils & turns	81.8%	81.8%

Supplementary Table 4. Heme-to-heme distances in both OmcZ^{Gs} and OmcZ2^{Mv}, related to Figure 3. Both iron-to-iron and minimum edge-to-edge distances were calculated.

	OmcZ^{Gs}		OmcZ^{Mv2}	
Unit (Å)	Iron-to-iron	Min edge-to-edge	Iron-to-iron	Min edge-to-edge
II - III	9.6	5.0	10.2	5.0
III - I	8.6	3.9	8.8	3.5
I - IV	9.5	5.2	9.8	5.0
IV - VII	10.0	4.8	9.6	4.0
VII - V	9.0	4.1	8.9	3.4
VII - VI	10.0	4.7	10.3	4.4
V - VIII	11.3	5.6	11.4	5.6
VIII - II	9.3	4.2	8.9	4.2

Supplementary Table 5. Summary of heme positions (top) and inter-heme regions (bottom) in both OmcZ^{Gs} and OmcZ2^{Mv}, related to Figure 3 & 4 Signal peptides are not included in residue positions. Heme binding motifs are not included in the inter-heme regions. Expanded inter-heme regions in OmcZ2^{Mv} are highlighted in red.

No. Heme	OmcZ^{Gs}	OmcZ2^{Mv}
Begin-I	25	33
I-II	22	26
II-III	17	12
III-IV	15	48
IV-V	42	39
V-VI	31	27
VI-VII	10	67
VII-VIII	38	36
VIII-end	6	5

Posi. Heme	OmcZ^{Gs}	OmcZ2^{Mv}
I	26-30	34-38
II	53-69	65-69
III	87-91	82-86
IV	107-111	135-139
V	154-158	179-183
VI	190-194	211-215
VII	205-209	280-284
VIII	248-252	321-325

Supplementary Table 6. Statistics of resolution estimates and model validations, related to

Resolution Estimates

Metric	Masked (Å)	Unmasked (Å)
Using map alone (d99)	3.84	3.70
Overall Biso	60.00	60.00
d_model	3.70	3.70
d_model (B factors = 0)	4.10	4.10
FSC (model) = 0	3.14	3.20
FSC (model) = 0.143	3.23	3.32
FSC (model) = 0.5	3.65	25.79
Resolution range (Å)	3.0-3.6	
Mask smoothing radius (Å)	6.52	

Model Validation Statistics

Chains	27
Atoms (Hydrogens)	8589 (0)
Residues	990
Ligands	HEC: 24
RMSD bond lengths (Å)	0.024
RMSD bond angles (°)	2.160
MolProbity score	2.85
Clash score	22.84
Ramachandran favored (%)	90.85
Ramachandran allowed (%)	8.94
Ramachandran outliers (%)	0.20
Rama-Z (whole / helix / sheet / loop)	-3.91 / -3.32 / NA / -2.67
Rotamer outliers (%)	4.17
CaBLAM outliers (%)	4.91
Occupancy mean	1.00
B-factor (mean / min / max)	117.35 / 30.00 / 179.26
CC (mask / box / peaks / volume)	0.85 / 0.73 / 0.52 / 0.85

Supplementary References

1. Becker, K. W., Elling, F. J., Schröder, J. M., Lipp, J. S., Goldhammer, T., Zabel, M., Elvert, M., Overmann, J., & Hinrichs, K. U. (2018). Isoprenoid quinones resolve the stratification of redox processes in a biogeochemical continuum from the photic zone to deep anoxic sediments of the Black Sea. Applied and Environmental Microbiology, *84*, e02736-17. https://doi.org/10.1128/AEM.02736-17.
