# Supplementary material for: Electrode-associated growth reveals structure of OmcZ-type cytochrome nanowires in anaerobic methanotrophic archaea
Source: Nat Commun. 2026 Nov 9;17:11117. doi: 10.1038/s41467-026-78976-x (PMC13748539; doi:10.1038/s41467-026-78976-x)
Supplement: Supplementary file 2 [file 41467_2026_78976_MOESM2_ESM.pdf]

Description of Additional Supplementary Files:

Supplementary Data 1: contains an annotation of the central metabolism of the MAGs with a focus on '*Ca*. M. vercellensis'.

Supplementary Data 2: contains the analyzed protein mass spectrometry data.
