# Supplementary material for: Electrode-associated growth reveals structure of OmcZ-type cytochrome nanowires in anaerobic methanotrophic archaea
Source: Nat Commun. 2026 Nov 9;17:11117. doi: 10.1038/s41467-026-78976-x (PMC13748539; doi:10.1038/s41467-026-78976-x)
Supplement: Supplementary file 3 [file 41467_2026_78976_MOESM5_ESM.pdf]

nature portfolio

Corresponding author(s): Cornelia Welte
Nikhil Malvankar

Last updated by author(s): Sep 10, 2026

Reporting Summary

Nature Portfolio wishes to improve the reproducibility of the work that we publish. This form provides structure for consistency and transparency in reporting. For further information on Nature Portfolio policies, see our Editorial Policies and the Editorial Policy Checklist.

Statistics

For all statistical analyses, confirm that the following items are present in the figure legend, table legend, main text, or Methods section.

n/a	Confirmed	
☐	☒	The exact sample size (n) for each experimental group/condition, given as a discrete number and unit of measurement
☐	☒	A statement on whether measurements were taken from distinct samples or whether the same sample was measured repeatedly
☐	☒	The statistical test(s) used AND whether they are one- or two-sided<br>*Only common tests should be described solely by name; describe more complex techniques in the Methods section.*
☒	☐	A description of all covariates tested
☒	☐	A description of any assumptions or corrections, such as tests of normality and adjustment for multiple comparisons
☐	☒	A full description of the statistical parameters including central tendency (e.g. means) or other basic estimates (e.g. regression coefficient) AND variation (e.g. standard deviation) or associated estimates of uncertainty (e.g. confidence intervals)
☒	☐	For null hypothesis testing, the test statistic (e.g. F, t, r) with confidence intervals, effect sizes, degrees of freedom and P value noted<br>*Give P values as exact values whenever suitable.*
☒	☐	For Bayesian analysis, information on the choice of priors and Markov chain Monte Carlo settings
☒	☐	For hierarchical and complex designs, identification of the appropriate level for tests and full reporting of outcomes
☒	☐	Estimates of effect sizes (e.g. Cohen's d, Pearson's r), indicating how they were calculated

Our web collection on statistics for biologists contains articles on many of the points above.

Software and code

Policy information about availability of computer code

Data collection	MultiTrace 4 software vs. 4.5.3920 on EmStat4 LR potentiostat
Data analysis	MultiTrace 4 software vs. 4.5.3920 on EmStat4 LR potentiostat<br>Filtlong v0.2.1 https://github.com/rrwick/Filtlong<br>Flye v2.8.3 citation 62: Kolmogorov, M., Bickhart, D.M., Behsaz, B., Gurevich, A., Rayko, M., Shin, S.B., Kuhn, K., Yuan, J., Polevikov, E., Smith, T.P.L., et al. (2020). metaFlye: scalable long-read metagenome assembly using repeat graphs. Nat Methods 17, 1103–1110. https://doi.org/10.1038/s41592-020-00971-x.<br>racon v1.4.21 citation 63: Vaser, R., Sović, I., Nagarajan, N., and Šikić, M. (2017). Fast and accurate de novo genome assembly from long uncorrected reads. Genome Res. 27, 737–746. https://doi.org/10.1101/gr.214270.116.<br>pilon5 v1.2.4 citation 64: Walker, B.J., Abeel, T., Shea, T., Priest, M., Abouelliel, A., Sakthikumar, S., Cuomo, C.A., Zeng, Q., Wortman, J., Young, S.K., et al. (2014). Pilon: An Integrated Tool for Comprehensive Microbial Variant Detection and Genome Assembly Improvement. PLoS ONE 9, e112963. https://doi.org/10.1371/journal.pone.0112963.<br>Aviary v0.9.0 https://github.com/rhysnewell/aviary<br>dRep v3.4.2 citation 65: Olm, M.R., Brown, C.T., Brooks, B., and Banfield, J.F. (2017). dRep: a tool for fast and accurate genomic comparisons that enables improved genome recovery from metagenomes through de-replication. The ISME Journal 11, 2864–2868. https://doi.org/10.1038/ismej.2017.126.<br>CheckM2 citation 66: Chklovski, A., Parks, D.H., Woodcroft, B.J., and Tyson, G.W. (2023). CheckM2: a rapid, scalable and accurate tool for assessing microbial genome quality using machine learning. Nat Methods 20, 1203–1212. https://doi.org/10.1038/s41592-023-01940-w.<br>CoverM v0.7.0. citation 67: Aroney, S.T.N., Newell, R.J.P., Nissen, J.N., Camargo, A.P., Tyson, G.W., and Woodcroft, B.J. (2025). CoverM: read alignment statistics for metagenomics. Bioinformatics 41, btaf147. https://doi.org/10.1093/bioinformatics/btaf147.

GTDB-Tk v2.4.0 (release R220) citation 68: Parks, D.H., Chuvochina, M., Waite, D.W., Rinke, C., Skarshewski, A., Chaumeil, P.-A., and Hugenholtz, P. (2018). A standardized bacterial taxonomy based on genome phylogeny substantially revises the tree of life. Nat Biotechnol 36, 996–1004. https://doi.org/10.1038/nbt.4229.
Prokka v1.14.6 citation 69: Seemann, T. (2014). Prokka: rapid prokaryotic genome annotation. Bioinformatics 30, 2068–2069. https://doi.org/10.1093/bioinformatics/btu153.
EggNOG v5.0 with eggNOG-mapper v2.1.12 citations 70, 71
SignalP v5 citation 76: Almagro Armenteros, J.J., Tsirigos, K.D., Sønderby, C.K., Petersen, T.N., Winther, O., Brunak, S., Von Heijne, G., and Nielsen, H. (2019). SignalP 5.0 improves signal peptide predictions using deep neural networks. Nat Biotechnol 37, 420–423. https://doi.org/10.1038/s41587-019-0036-z.
MEGA-X citation https://megasoftware.net/
iTOL citation 77: Letunic, I., and Bork, P. (2021). Interactive Tree Of Life (iTOL) v5: an online tool for phylogenetic tree display and annotation. Nucleic Acids Research 49, W293–W296. https://doi.org/10.1093/nar/gkab301.
PEAKS Studio X from Bioinformatics Solutions Inc., Waterloo, Canada
cryoSPARC citation 79: Punjani, A., Rubinstein, J.L., Fleet, D.J., and Brubaker, M.A. (2017). cryoSPARC: algorithms for rapid unsupervised cryo-EM structure determination. Nat Methods 14, 290–296. https://doi.org/10.1038/nmeth.4169.
Spring citation 80: Desfosses, A., Ciuffa, R., Gutsche, I., and Sachse, C. (2014). SPRING – An image processing package for single-particle based helical reconstruction from electron cryomicrographs. Journal of Structural Biology 185, 15–26. https://doi.org/10.1016/j.jsb.2013.11.003.
AlphaFold3 citation 81: Abramson, J., Adler, J., Dunger, J., Evans, R., Green, T., Pritzel, A., Ronneberger, O., Willmore, L., Ballard, A.J., Bambrick, J., et al. (2024). Accurate structure prediction of biomolecular interactions with AlphaFold 3. Nature 630, 493–500. https://doi.org/10.1038/s41586-024-07487-w.
ChimeraX citation 82: Pettersen, E.F., Goddard, T.D., Huang, C.C., Meng, E.C., Couch, G.S., Croll, T.I., Morris, J.H., and Ferrin, T.E. (2021). UCSF CHIMERAX: Structure visualization for researchers, educators, and developers. Protein Science 30, 70–82. https://doi.org/10.1002/pro.3943.
PHENIX citation 83: Adams, P.D., Afonine, P.V., Bunkóczi, G., Chen, V.B., Davis, I.W., Echols, N., Headd, J.J., Hung, L.-W., Kapral, G.J., Grosse-Kunstleve, R.W., et al. (2010). PHENIX : a comprehensive Python-based system for macromolecular structure solution. Acta Crystallogr D Biol Crystallogr 66, 213–221. https://doi.org/10.1107/S0907444909052925.
Coot citation 84: Emsley, P., and Cowtan, K. (2004). Coot : model-building tools for molecular graphics. Acta Crystallogr D Biol Crystallogr 60, 2126–2132. https://doi.org/10.1107/S0907444904019158.

For manuscripts utilizing custom algorithms or software that are central to the research but not yet described in published literature, software must be made available to editors and reviewers. We strongly encourage code deposition in a community repository (e.g. GitHub). See the Nature Portfolio guidelines for submitting code & software for further information.

Data

Policy information about availability of data

All manuscripts must include a data availability statement. This statement should provide the following information, where applicable:

- Accession codes, unique identifiers, or web links for publicly available datasets
- A description of any restrictions on data availability
- For clinical datasets or third party data, please ensure that the statement adheres to our policy

Sequencing data and MAGs have been deposited in the European Nucleotide Archive (ENA) under bioproject PRJEB98224 (https://www.ebi.ac.uk/ena/browser/view/PRJEB98224). The chromosome of Ca. M. vercellensis is deposited within the bioproject under accession 'GCA_988232875.1' whereas the associated plasmid has been published previously under bioproject PRJNA850006 as biosample SAMN31367896 (https://ncbi.nlm.nih.gov/nuccore/CP113316.1). Structural data has been deposited in the RCSB Protein Data Bank under accession 10RP (https://www.rcsb.org/structure/unreleased/10RP). Proteomic data is available on the PRIDE database under accession PXD072602 (https://www.ebi.ac.uk/pride/archive/projects/PXD072602). Source data are provided with this paper.

Research involving human participants, their data, or biological material

Policy information about studies with human participants or human data. See also policy information about sex, gender (identity/presentation), and sexual orientation and race, ethnicity and racism.

Reporting on sex and gender	not applicable
Reporting on race, ethnicity, or other socially relevant groupings	not applicable
Population characteristics	not applicable
Recruitment	not applicable
Ethics oversight	not applicable

Note that full information on the approval of the study protocol must also be provided in the manuscript.

Field-specific reporting

For a reference copy of the document with all sections, see nature.com/documents/nr-reporting-summary-flat.pdf

Life sciences study design

All studies must disclose on these points even when the disclosure is negative.

Sample size	For physiological measurements, biological triplicates were chosen; in some cases the material was not sufficient and biological duplicates were conducted. Micrographs show representative images of three experiments.
Data exclusions	No data were excluded
Replication	Bioelectrochemical data were replicable on general characteristics but not on the absolute produced current because the amount of biomass attached to the electrode could not be steered and varied between different experiments; that is why all individual experiments are reported in the paper, rather than just averages and deviation.
Randomization	Randomization was not relevant to this study.
Blinding	Blinding was not relevant in this study.

Reporting for specific materials, systems and methods

We require information from authors about some types of materials, experimental systems and methods used in many studies. Here, indicate whether each material, system or method listed is relevant to your study. If you are not sure if a list item applies to your research, read the appropriate section before selecting a response.

Materials & experimental systems

n/a	Involved in the study	
☒	☐	Antibodies
☒	☐	Eukaryotic cell lines
☒	☐	Palaeontology and archaeology
☒	☐	Animals and other organisms
☒	☐	Clinical data
☒	☐	Dual use research of concern
☒	☐	Plants

Methods

n/a	Involved in the study	
☒	☐	ChIP-seq
☒	☐	Flow cytometry
☒	☐	MRI-based neuroimaging

Plants

Seed stocks	Not applicable
Novel plant genotypes	Not applicable
Authentication	Not applicable
