# Supplementary material for: Electrode-associated growth reveals structure of OmcZ-type cytochrome nanowires in anaerobic methanotrophic archaea
Source: Nat Commun. 2026 Nov 9;17:11117. doi: 10.1038/s41467-026-78976-x (PMC13748539; doi:10.1038/s41467-026-78976-x)
Supplement: Supplementary file 4 [file 41467_2026_78976_MOESM6_ESM.pdf]

nature portfolio

Peer Review File

Electrode-associated growth reveals structure of OmcZ-type cytochrome nanowires in anaerobic methanotrophic archaea

Corresponding Author: Professor Cornelia Welte

This file contains all reviewer reports in order by version, followed by all author rebuttals in order by version.

Version 0:

Reviewer comments:

Reviewer #1

(Remarks to the Author)
The manuscript by Wissink et al. addresses a long-standing and significant research gap in the field of anaerobic methane oxidation (AOM) and electromicrobiology: whether Candidatus Methanoperedens can perform extracellular electron transfer (EET) via multiheme c-type cytochrome (MHC) nanowires. While previous genomic and transcriptomic evidence suggested this physiological trait, direct experimental proof has been lacking. This study provides clear physiological and molecular evidence supporting this mechanism.
The authors successfully employed BES to cultivate Ca. Methanoperedens under poised electrodes and obtained a near-axenic culture of a freshwater ANME, named Ca. Methanoperedens vercellensis. Obtaining such a near-axenic culture is highly impressive and represents a major technical achievement. This culture not only provides the first demonstration that ANME archaea can grow on electrodes, but also establishes a critical model system for mechanistic studies of nanowire-mediated EET in archaea.
Key physiological parameters, including doubling time, electron carrier preference, and substrate specificity, are clearly determined. The 3.3 Å cryo-EM structure of polymerized OmcZ-type cytochrome nanowires is a particular strength, revealing structural features optimized for long-range EET. Furthermore, the analysis of OmcZ-type nanowire distribution across phylogenetically diverse ANME lineages provides important insights into the evolutionary conservation and ecological relevance of this EET mechanism, substantially advancing our understanding of how ANME archaea may mediate extracellular electron transfer in natural environments.
Overall, this work represents a major advance in both the AOM and electromicrobiology fields. The experimental design is sound, the methodologies are appropriate and state-of-the-art, and the conclusions are supported by the data. The manuscript is well-written and easy to follow. I can see the limitations of the study that the culture is not absolutely axenic, which could potentially interfere with physiological characterization, and that the limited biomass precluded certain characterizations of nanowire properties (e.g., direct conductivity measurements). These limitations have been recognized by the authors in the manuscript. Nevertheless, I consider the evidence presented in this study to be a substantial and important advance. The work provides sufficient and compelling evidence to significantly move our understanding of nanowire-mediated EET in ANME forward. I only have some minor comments for the authors to address:
1) In the authors' previous work (doi.org/10.1038/s41467-024-45758-2), Ca. Methanoperedens sp. BLZ2 was used for EET studies, and MHC nanowires were also proposed. The current study uses a different species, Ca. Methanoperedens vercellensis. Could the authors please explain the rationale for using a different strain and discuss any potential differences or similarities between the two strains regarding their EET-dependent growth and EET mechanisms, particularly the MHC nanowires involved? I recommend adding a brief discussion comparing the MHC nanowires for EET between these two Ca. Methanoperedens species in the Discussion section.
2) Although I am convinced by the calculation of doubling time based on current production, it would be better to provide "real" growth data, such as biomass concentration (e.g., protein content) or direct cell counting, to further substantiate the reported doubling time.
3) Line 118-119: I did not find the data for negative-stain TEM images that are referenced in the text. Please ensure these are included.
4) Lines 150-166: I appreciate the authors' effort to test different electron acceptors with the inoculum and electrode-accumulated culture. However, I found the ethane oxidation assay to be of limited relevance to the main focus of this study, which centers on methane-dependent EET and nanowire mechanisms. While the negative result is interesting, its inclusion somewhat distracts from the core narrative. I suggest either moving this data to the supplementary materials or providing a clearer justification in the text for why testing ethane oxidation is important for understanding the EET physiology of Ca.

Methanoperedens vercellensis.
5) Line 165-166: Could the authors specify the potential nitrite-scavenging partners present in the inoculum? A brief discussion of this point would help readers interpret the observed metabolic interactions.

Reviewer #2

(Remarks to the Author)
Wissink et al presented an interesting manuscript on an OmcZ-type nanowire from a newly identified species ‘Ca. M. vercellensis’. This microorganism predates on methane for its electron source, thus the name Methanoperedens. Methane is a green-house gas involved in global warming, making the paper potentially interesting to the general audience of Nature Communications.

Cytochrome nanowires are conductive protein filaments produced by bacteria and archaea to transport electrons outside the cell. In this manuscript, the authors used bioelectrochemical systems to grow Ca. M. vercellensis to near-axenic cultures. In this way, they were able to harvest nanowires from the bacteria and solve a cryo-EM structure of this nanowire. They also deduced key physiological properties of Ca. M. vercellensis in this electrode-grown culture.

Although the cryo-EM structure was properly done and the helical solution was correct, the resolution of this structure is substantially over-claimed. Despite the main-chain densities look continuous and well-shaped after AI enhancement, the side-chain densities fall well short of a 3.3 Å cryo-EM structure. Many of the aromatic side-chains that are close to each other are not fully resolved from each other, suggesting the resolution of this structure is poorer than 4.0 Å. Indeed, if there is no homologue of this protein previously solved, it is virtually impossible to trace the main-chain unambiguously, given its limited secondary structure content.

One last comment on the manuscript is that the details for the cryo-EM refinement and the helical solution were very limited. A typical helical cryo-EM paper, for example, should show the Fourier transform of the 2D classes to index the layer lines. It may also show the approximate selection rule to illustrate the helical solution. Such details need to appear at least in the Methods section.

I suggest the authors address these points and either improve the resolution of the structure or tone down relevant claims drawn on the reported resolution.

Reviewer #3

(Remarks to the Author)
The manuscript “Electrode-associated growth reveals structure of OmcZ-type cytochrome nanowires in anaerobic methanotrophic archaea” by Wissink et al. presents a comprehensive study of an electrode-enriched culture of a freshwater ANME archaeon (Ca. Methanoperedens) and provides valuable insights into its physiology. The authors report enrichment of Ca. Methanoperedens to high relative abundance and characterize several physiological features of the culture. A particularly interesting aspect is the purification and structural determination (3.3 Å resolution) of an OmcZ homolog from this enrichment.
OmcZ-type cytochromes have been extensively studied in bacterial systems such as Geobacter, where they play key roles in extracellular electron transfer (EET). Homologs are also present in and expressed by ANME lineages, suggesting they may contribute to archaeal EET. In this context, the structural data presented here are potentially significant and advance our understanding of archaeal OmcZ-like proteins and their possible assembly into higher-order conductive structures.
While the study addresses an important topic and presents potentially valuable data, several aspects of the structural and functional interpretation would benefit from additional clarification and a more cautious framing of the physiological implications. In particular, some statements regarding the role of the proposed nanowires would benefit from being more explicitly linked to the available experimental evidence. Although the authors appropriately acknowledge some limitations, the comments below are intended to help strengthen the rigor, clarity, and interpretation of the study.

1) The authors performed an anoxic BES experiment to investigate methanotrophy by ANME-2d archaea. However, the second most abundant organism detected (6.2%) belongs to the genus Bosea, which is described as strictly aerobic (line 124). Please clarify how the presence and persistence of this organism can be reconciled with the reported anoxic conditions in the BES system and whether it could impact the studied methanotrophy.

2) The interpretation of the purified nanowire-associated protein fraction based on mass spectrometry and structural assignment would benefit from further clarification.

a. The authors report purification of putative nanowire-forming cytochromes from the culture medium (rather than from the electrode-associated biofilm). Please clarify how this fraction relates to the physiological context of EET between cells and the electrode.
b. The gene identifier used for the reported protein homologs is not publicly accessible, which limits independent verification. I encourage the authors to provide standard, publicly available gene/protein accessions (e.g., GenBank/RefSeq/IMG) to improve reproducibility.
c. The ~70 kDa band detected by Coomassie and heme staining is assigned to an OmcZ homolog (OmcZ2MV) as the most abundant cytochrome; however, the mass spectrometry analysis (Sup Fig. 3B) identifies this protein with only three unique peptides and ~8% sequence coverage, whereas several other proteins in the same fraction appear to have stronger peptide

support. Please clarify how the protein assignment (and “most abundant” designation) was prioritized under these conditions. In addition, the mass spectrometry results would benefit from clearer presentation (e.g., the PTMs listed in the table are not explained in the legend). It would also be helpful to clarify whether any of the reported modifications relate to c-type cytochrome/heme maturation, and whether heme-modified peptides were detected (e.g., evidence consistent with covalently attached heme on CXXCH-containing peptides).
d. The manuscript states that the organism encodes 39 c-type cytochromes. Given this diversity, please clarify whether OmcZ2 is the only cytochrome detected in the purified band, and how its assignment as the structural component is distinguished from other homologous cytochromes. In particular, could a related cytochrome homolog also be consistent with the reported 3.3 Å model?
e. Because the enrichment originated from a mixed microbial biofilm community that previously included electroactive microbes such as Geobacter spp., please clarify whether the mass spectrometry search database included representative sequences from these organisms. Could residual proteins from such community members potentially contribute to the observed band?
f. The detection of peptides spanning the full-length OmcZ sequence in the culture medium is interpreted as evidence for nanowire-forming and cleaved forms of the protein (Sup Fig 3A). Please clarify why this signal could not also reflect the unprocessed precursor form of OmcZ (or a mixture of processed and unprocessed forms).

3) The physiological interpretation of the OmcZ homolog, based primarily on bioinformatic and structural comparisons, would benefit from a more cautious presentation unless supported by additional experimental evidence. In particular, additional discussion of the proposed mechanism of OmcZ maturation and export in ANME archaea would strengthen the manuscript. Current models for OmcZ-like nanowire assembly in bacteria suggest that the protein undergoes maturation and cleavage prior to polymerization and export, potentially involving type IV pilus/secretion-associated systems. In this context, it would be valuable for the authors to discuss how analogous processes might occur in ANME cells, and how the proposed nanowire structures could facilitate electron transfer from intracellular methane oxidation pathways to extracellular electron acceptors such as electrodes.

4) I wonder whether relative abundance alone is the best proxy for microbial load or electrochemical activity. In Ouboter et al. (2024), ANME-2d enrichment reached ~82%, whereas methane-dependent current accounted for ~91–93% of the signal. In the present study, ANME-2d abundance increases to 95.8%, but methane-dependent current increases only marginally (to ~97%). Does this simply reflect normal experimental variability, or does it suggest a more complex relationship between relative abundance and current production?

5) The manuscript reports that Geobacter relative abundance increased from 8.5% in the inoculum to ~18% in the enrichment biofilm after 67 days, before becoming undetectable at the later enrichment stage (119 days) (Lines 116–118). In this context, the explanation that the observed signal originates solely from residual or necromass DNA (Line 120) may not fully account for the earlier enrichment dynamics.

Version 1:

Reviewer comments:

Reviewer #2

(Remarks to the Author)
The manuscript, as it stands now, satisfies all my previous concerns. It is ready for publication.

One minor issue: there is a typo in the Supplementary Figure S6, panel D, the labels of the layer-lines should read l=-2,-1,0,+1,+2 instead of n=…

Reviewer #3

(Remarks to the Author)
Thank you for the opportunity to review the revision. The manuscript has improved and addresses some of my earlier concerns, including the issue of potential aerobic contamination in the enrichment and the clarification that the filaments were purified from the electrode-associated biofilm rather than the culture medium (as described differently in the previous version).
One point I remain uncertain about is the strength of the claims that OmcZ2Mv forms “highly conductive” nanowires. In the section “Structural features optimized for high conductivity,” the authors infer enhanced conductivity/EET primarily from comparative structural features (e.g., inter-heme spacing/packing and surrounding interactions). These are interesting observations; however, it remains unclear whether the proteomics/MS analysis provides peptide-level evidence for covalent c-type heme attachment (e.g., heme-adduct mass shifts on a CXXCH-containing peptide), even though a peptide containing the CXXCH heme-binding motif was detected. The band is heme-positive by staining and the structural work is compelling, but in the absence of clearly documented MS evidence of covalent heme attachment and direct conductivity measurements, some of the “high conductivity/optimized” language appears stronger than what is directly supported.
Relatedly, my question about whether other Ca. Methanoperedens c-type cytochromes were detected in the excised band/fraction is only partially addressed. A clear summary stating whether any additional c-type cytochromes were detected (even at low abundance), or explicitly that none were detected, would be helpful. The response indicates that only an OmcZ homolog was detected, but it remains unclear whether additional (non-OmcZ) c-type cytochromes were present at lower

abundance or absent.
Modest toning down or qualification of the ‘optimized for high conductivity’/‘highly conductive’ wording would help align the conclusions with the evidence presented.
Given the central role proposed for multiheme cytochrome c proteins (including OmcZ homologs) in ANME extracellular electron transfer, I encourage the authors to clarify/temper these statements where needed and ensure that the wording reflects the level of direct support.

Open Access This Peer Review File is licensed under a Creative Commons Attribution 4.0 International License, which permits use, sharing, adaptation, distribution and reproduction in any medium or format, as long as you give appropriate credit to the original author(s) and the source, provide a link to the Creative Commons license, and indicate if changes were made.
In cases where reviewers are anonymous, credit should be given to 'Anonymous Referee' and the source.
The images or other third party material in this Peer Review File are included in the article’s Creative Commons license, unless indicated otherwise in a credit line to the material. If material is not included in the article’s Creative Commons license and your intended use is not permitted by statutory regulation or exceeds the permitted use, you will need to obtain permission directly from the copyright holder.
To view a copy of this license, visit https://creativecommons.org/licenses/by/4.0/

Reviewer #1 (Remarks to the Author):

The manuscript by Wissink et al. addresses a long-standing and significant research gap in the field of anaerobic methane oxidation (AOM) and electromicrobiology: whether Candidatus Methanoperedens can perform extracellular electron transfer (EET) via multiheme c-type cytochrome (MHC) nanowires. While previous genomic and transcriptomic evidence suggested this physiological trait, direct experimental proof has been lacking. This study provides clear physiological and molecular evidence supporting this mechanism.
The authors successfully employed BES to cultivate Ca. Methanoperedens under poised electrodes and obtained a near-axenic culture of a freshwater ANME, named Ca. Methanoperedens vercellensis. Obtaining such a near-axenic culture is highly impressive and represents a major technical achievement. This culture not only provides the first demonstration that ANME archaea can grow on electrodes, but also establishes a critical model system for mechanistic studies of nanowire-mediated EET in archaea.
Key physiological parameters, including doubling time, electron carrier preference, and substrate specificity, are clearly determined. The 3.3 Å cryo-EM structure of polymerized OmcZ-type cytochrome nanowires is a particular strength, revealing structural features optimized for long-range EET. Furthermore, the analysis of OmcZ-type nanowire distribution across phylogenetically diverse ANME lineages provides important insights into the evolutionary conservation and ecological relevance of this EET mechanism, substantially advancing our understanding of how ANME archaea may mediate extracellular electron transfer in natural environments.

Overall, this work represents a major advance in both the AOM and electromicrobiology fields. The experimental design is sound, the methodologies are appropriate and state-of-the-art, and the conclusions are supported by the data. The manuscript is well-written and easy to follow. I can see the limitations of the study that the culture is not absolutely axenic, which could potentially interfere with physiological characterization, and that the limited biomass precluded certain characterizations of nanowire properties (e.g., direct conductivity measurements). These limitations have been recognized by the authors in the manuscript. Nevertheless, I consider the evidence presented in this study to be a substantial and important advance. The work provides sufficient and compelling evidence to significantly move our understanding of nanowire-mediated EET in ANME forward. I only have some minor comments for the authors to address:

1) In the authors' previous work (doi.org/10.1038/s41467-024-45758-2), Ca.

Methanoperedens sp. BLZ2 was used for EET studies, and MHC nanowires were also proposed. The current study uses a different species, Ca. Methanoperedens vercellensis. Could the authors please explain the rationale for using a different strain and discuss any potential differences or similarities between the two strains regarding their EET-dependent growth and EET mechanisms, particularly the MHC nanowires involved? I recommend adding a brief discussion comparing the MHC nanowires for EET between these two Ca. Methanoperedens species in the Discussion section.

Our previous paper on EET in Methanoperedens (doi.org/10.1038/s41467-024-45758-2), used the same strain as this study, Methanoperedens isolated from Vercelli rice fields, now named Methanoperedens vercellensis. Maybe confusion arose when it was re-named in this manuscript which is described in line 87. The BLZ2 strain that the referee mentions was used neither in this nor the previous article and is indeed a different strain (now named Methanoperedens carboxydivorans) that we use for different types of research. It has not been mentioned in this or the previous EET manuscript, so we refrain from comparing their genomes or physiology.

2) Although I am convinced by the calculation of doubling time based on current production, it would be better to provide "real" growth data, such as biomass concentration (e.g., protein content) or direct cell counting, to further substantiate the reported doubling time.

We acknowledge this limitation of our study; unfortunately, due to the need of sacrificial sampling of entire electrodes and the long incubation times of the electrogenic growth it was not possible to collect the data the referee is suggesting. If the cells were dispersed in the anolyte we could just take samples throughout the experiments but as they form tight biofilms with low biomass content, we were not able to determine cell counts or protein content. We did perform end-point protein measurements between different transfers and saw that new protein was clearly made, however we think that these data may be misleading to the readers as they do not represent a 'growth' estimate, but rather show that indeed new cellular material has been made in a qualitative sense.

3) Line 118-119: I did not find the data for negative-stain TEM images that are referenced in the text. Please ensure these are included.

Please refer to Fig. 3B for the negative-stain TEM image mentioned here. To increase clarity in particular with regard to the differentiation between *Geobacter* and *Ca*. Methanoperedens, we have also added another negative-stain TEM image in the

supplementary data (Figure S3) that provides a view on an S-layer containing archaeal cell, representing *Ca*. Methanoperedens identified by the characteristic archaeal surface structure, with protruding nanowire-like appendages.

4) Lines 150-166: I appreciate the authors' effort to test different electron acceptors with the inoculum and electrode-accumulated culture. However, I found the ethane oxidation assay to be of limited relevance to the main focus of this study, which centers on methane-dependent EET and nanowire mechanisms. While the negative result is interesting, its inclusion somewhat distracts from the core narrative. I suggest either moving this data to the supplementary materials or providing a clearer justification in the text for why testing ethane oxidation is important for understanding the EET physiology of Ca. Methanoperedens vercellensis.

These experimental data have been moved to the supplement as suggested.

5) Line 165-166: Could the authors specify the potential nitrite-scavenging partners present in the inoculum? A brief discussion of this point would help readers interpret the observed metabolic interactions.

We appreciate this suggestion by the referee. We have carefully assessed the nitrite reduction potential in the reported MAGs and have added this analysis to Supplementary Data 2 as a separate tab to report the nitrite and nitrate reductases encoded. It became clear that indeed the four main taxa encoding nitrite reductases where present in the inoculum but absent in the BESs. Intriguingly, Ca. Methanoperedens also encodes a nitrite reductase as we reported previously (Arshad et al., 2015) but it doesn't seem to be able to turnover enough nitrite to counteract nitrite poisoning; the reason for this remains unknown. The respective bacterial taxa potentially involved in nitrite scavenging have been added to the text:

Lines 167-168 "Supplementary Data 2; genomes encoding dissimilatory nitrite reductases in the inoculum but absent in the BESs were Gallionellaceae, Geobacter, Casimicrobiaceae, Phycisphaerales".

Reviewer #2 (Remarks to the Author):

Wissink et al presented an interesting manuscript on an OmcZ-type nanowire from a newly identified species 'Ca. M. vercellensis'. This microorganism predates on methane for its

electron source, thus the name Methanoperedens. Methane is a green-house gas involved in global warming, making the paper potentially interesting to the general audience of Nature Communications.

Cytochrome nanowires are conductive protein filaments produced by bacteria and archaea to transport electrons outside the cell. In this manuscript, the authors used bioelectrochemical systems to grow Ca. M. vercellensis to near-axenic cultures. In this way, they were able to harvest nanowires from the bacteria and solve a cryo-EM structure of this nanowire. They also deduced key physiological properties of Ca. M. vercellensis in this electrode-grown culture.

Although the cryo-EM structure was properly done and the helical solution was correct, the resolution of this structure is substantially over-claimed. Despite the main-chain densities look continuous and well-shaped after AI enhancement, the side-chain densities fall well short of a 3.3 Å cryo-EM structure. Many of the aromatic side-chains that are close to each other are not fully resolved from each other, suggesting the resolution of this structure is poorer than 4.0 Å. Indeed, if there is no homologue of this protein previously solved, it is virtually impossible to trace the main-chain unambiguously, given its limited secondary structure content.

We thank the reviewer for raising this point. We would like to clarify that no AI-based map enhancement tools were employed at any stage of our reconstruction pipeline. The continuous main-chain densities therefore reflect experimental data rather than artifacts of post-processing sharpening or denoising.

However, we acknowledge that the map quality could benefit from further optimization. We have therefore re-processed the dataset and obtained a reconstruction at 3.29 Å resolution. The gold-standard Fourier shell correlation (GSFSC) curves (Supplementary Fig. S5J) confirm a reliable global resolution estimate. We also include the particle orientation distribution (Fig. S6A) and a local-resolution analysis shown as a resolution-colored surface (Fig. S6B), the latter demonstrating the majority of the map reaches local resolutions better than 4.0 Å. Furthermore, a homologous structure determined in our previous study (*Gu et al., Nat Micro., 2023*) serves as an independent reference for model validation. Although the secondary structure content of this protein is limited, unambiguous fitting of side-chain densities for histidine and the cysteine residues coordinating the heme cofactors supports the reliability of our atomic model.

One last comment on the manuscript is that the details for the cryo-EM refinement and the helical solution were very limited. A typical helical cryo-EM paper, for example, should show the Fourier transform of the 2D classes to index the layer lines. It may also show the approximate selection rule to illustrate the helical solution. Such details need to appear at least in the Methods section.

We thank the reviewer for this constructive suggestion. We have included the requested information in Supplementary Figure S5CD. We computed the power spectrum from the selected 2D class average (Fig. S6C) using the Average Power Spectra job in cryoSPARC (v4.7.1), which reveals discrete layer lines equally spaced along the meridian and symmetric about the equator (Fig. S6D). As established by Cochran, Crick & Vand (1952), such discrete, equally spaced, equator-symmetric layer lines constitute the reciprocal-space signature of a helical structure, with the axial repeat encoded in their spacing. We subsequently determined the helical parameters using cryoSPARC's symmetry search utility, yielding a helical symmetry of rise = 58.0 Å and twist = 159.5°. These values were applied in the subsequent helical refinement.

Additionally, we have revised the following descriptions in the Methods section:

line 691-692

"…in cryoSPARC. Helical symmetry parameters, including helical rise and twist, were determined using as a helical rise of 58.0 Å and a twist of 159.5°. Map FSC …"

I suggest the authors address these points and either improve the resolution of the structure or tone down relevant claims drawn on the reported resolution.

As discussed in the above response, we have now improved the resolution as per the referee's suggestion.

Reviewer #3 (Remarks to the Author):

The manuscript "Electrode-associated growth reveals structure of OmcZ-type cytochrome

nanowires in anaerobic methanotrophic archaea" by Wissink et al. presents a comprehensive study of an electrode-enriched culture of a freshwater ANME archaeon (Ca. Methanoperedens) and provides valuable insights into its physiology. The authors report enrichment of Ca. Methanoperedens to high relative abundance and characterize several physiological features of the culture. A particularly interesting aspect is the purification and structural determination (3.3 Å resolution) of an OmcZ homolog from this enrichment. OmcZ-type cytochromes have been extensively studied in bacterial systems such as Geobacter, where they play key roles in extracellular electron transfer (EET). Homologs are also present in and expressed by ANME lineages, suggesting they may contribute to archaeal EET. In this context, the structural data presented here are potentially significant and advance our understanding of archaeal OmcZ-like proteins and their possible assembly into higher-order conductive structures.
While the study addresses an important topic and presents potentially valuable data, several aspects of the structural and functional interpretation would benefit from additional clarification and a more cautious framing of the physiological implications. In particular, some statements regarding the role of the proposed nanowires would benefit from being more explicitly linked to the available experimental evidence. Although the authors appropriately acknowledge some limitations, the comments below are intended to help strengthen the rigor, clarity, and interpretation of the study.

1) The authors performed an anoxic BES experiment to investigate methanotrophy by ANME-2d archaea. However, the second most abundant organism detected (6.2%) belongs to the genus Bosea, which is described as strictly aerobic (line 124). Please clarify how the presence and persistence of this organism can be reconciled with the reported anoxic conditions in the BES system and whether it could impact the studied methanotrophy.

We thank the referee for this observation. We think that *Bosea* likely benefits from trace oxygen amounts leaking into the BES, in addition to exudates or necromass from '*Ca*. Methanoperedens'. We have added a sentence reflecting these hypotheses to line 125: "It likely thrives on trace oxygen amounts leaking into the BES, as well as carbon exudates or necromass from '*Ca*. M. vercellensis'."

2) The interpretation of the purified nanowire-associated protein fraction based on mass spectrometry and structural assignment would benefit from further clarification.

a. The authors report purification of putative nanowire-forming cytochromes from the culture medium (rather than from the electrode-associated biofilm). Please clarify how this fraction relates to the physiological context of EET between cells and the electrode.

We apologize for the confusion. All filaments were purified from the biofilm. We corrected the methods section in line 636 accordingly.

b. The gene identifier used for the reported protein homologs is not publicly accessible, which limits independent verification. I encourage the authors to provide standard, publicly available gene/protein accessions (e.g., GenBank/RefSeq/IMG) to improve reproducibility.

We thank the referee for this input; the raw reads are already available on ENA as reported in the data availability paragraph; the MAGs are available for the referees on figshare under https://figshare.com/s/310b16af6e6eb443b46e (doi:10.6084/m9.figshare.32731260); they will be added to the ENA project folder as soon as the peer review is concluded because once submitted, nothing can be changed any more so we prefer to wait until all comments of referees have been addressed.

The figshare link has been added to the Resource availability paragraph.

c. The ~70 kDa band detected by Coomassie and heme staining is assigned to an OmcZ homolog (OmcZ2MV) as the most abundant cytochrome; however, the mass spectrometry analysis (Sup Fig. 3B) identifies this protein with only three unique peptides and ~8% sequence coverage, whereas several other proteins in the same fraction appear to have stronger peptide support. Please clarify how the protein assignment (and "most abundant" designation) was prioritized under these conditions. In addition, the mass spectrometry results would benefit from clearer presentation (e.g., the PTMs listed in the table are not explained in the legend). It would also be helpful to clarify whether any of the reported modifications relate to c-type cytochrome/heme maturation, and whether heme-modified peptides were detected (e.g., evidence consistent with covalently attached heme on CXXCH-containing peptides)

That the target protein is the most abundant protein can be inferred from both the abundance of the observed peptides and their spectral counts, which were significantly higher than those of all other background proteins. However, we believe the apparently low sequence coverage is the result of two factors. First, the nanowire-forming region is substantially smaller than the full annotated protein sequence because both the N-terminal signal peptide (MKNFKIVIPALLLFAVLVGIGI, see reviewer image below) and the C-terminal domains are cleaved, with the latter detected in the

proteomics experiment (Supplementary Data 3, first pane). Nevertheless, the sequence coverage was calculated against the full-length protein sequence.

When considering the nanowire forming region only, and enabling semi-specific cleavage, a highly abundant N-terminal peptide (cleaved after I), together with another short sequence fragment could also be detected, increasing the number of unique sequence fragments to five (see image below) and the sequence coverage to approximately 20%. The remaining nanowire-forming sequence region either spans heme-binding motifs or generates very short tryptic fragments that are difficult to observe under conventional proteomics conditions. For example, the protein contains eight closely spaced CXXCH heme motifs, which are expected to increase structural stability and resistance to denaturation, thereby reducing accessibility for efficient proteolytic digestion. In addition, the attached heme groups were apparently not fully removed under the applied reducing conditions (only one heme sequence spanning peptide was detected), resulting in large and hydrophobic peptide fragments that were not readily accessible for proteomic detection.

However, as mentioned above, the detected peptides showed the highest peptide areas and largest number of spectral counts compared to all other background proteins. Manual inspection of the LC-MS traces further confirmed that the majority of visually abundant peptide features originated either from either the target protein, trypsin, or singly charged background ions.

Regarding modifications, we included deamidation, oxidation, and carbamidomethylation of cysteines, the latter introduced during reduction and alkylation with iodoacetamide. No significant evidence for glycosylation was detected.

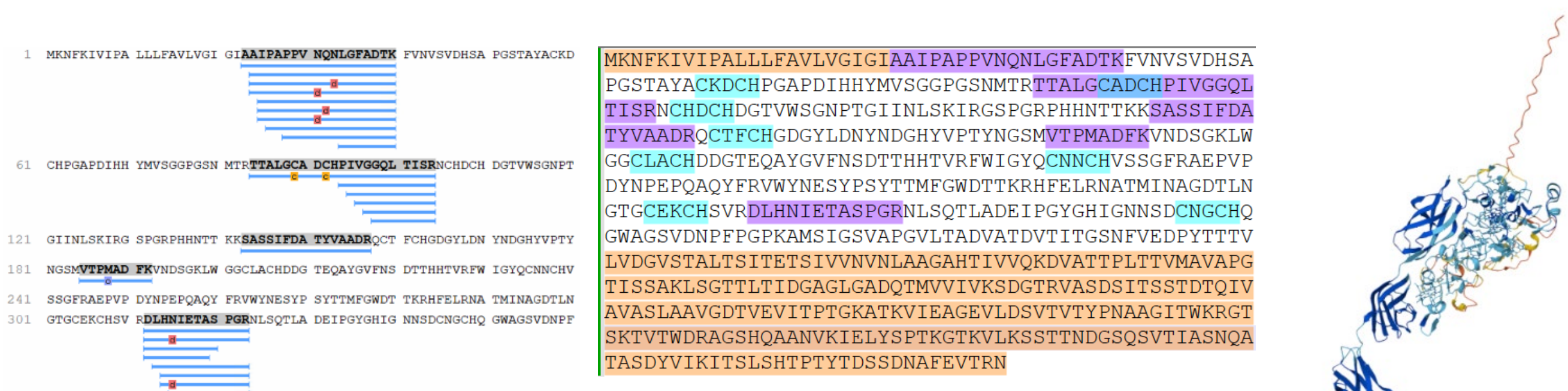

Reviewer figure: The left panel shows the expanded sequence coverage when considering only the nanowire-forming region, taking into account cleavage of the N-terminal signal peptide (long extended structure at the top of the AlphaFold model, right panel) and cleavage of the C-terminal domains (dark blue regions in the AlphaFold model, right panel). The middle panel shows the amino acid sequence, the positions of the heme groups (turquoise), and the detected peptide sequences (purple),

which are primarily located between the heme-binding regions. The orange regions indicate the cleaved sequence segments that were presumably not present in the excised gel band.

d. The manuscript states that the organism encodes 39 c-type cytochromes. Given this diversity, please clarify whether OmcZ2 is the only cytochrome detected in the purified band, and how its assignment as the structural component is distinguished from other homologous cytochromes. In particular, could a related cytochrome homolog also be consistent with the reported 3.3 Å model?

Yes, this one was the only detected OmcZ homolog in the purified band. The amino acid identity to a second OmcZ 'homolog' encoded in the Ca. Methanoperedens genome is only 33% identical to this protein so they are clearly distinguished from one another.

e. Because the enrichment originated from a mixed microbial biofilm community that previously included electroactive microbes such as Geobacter spp., please clarify whether the mass spectrometry search database included representative sequences from these organisms. Could residual proteins from such community members potentially contribute to the observed band?

The database searching was performed using a metagenome-derived sequence database of the microbial community; therefore, any remaining microbial proteins were included in the search space and would have been detected. At the time of sampling, the metagenome sequencing did not detect *Geobacter* sequences, indicating that they were not present on the electrode any longer, in addition to the absence of *Geobacter* in the proteomics dataset.

f. The detection of peptides spanning the full-length OmcZ sequence in the culture medium is interpreted as evidence for nanowire-forming and cleaved forms of the protein (Sup Fig 3A). Please clarify why this signal could not also reflect the unprocessed precursor form of OmcZ (or a mixture of processed and unprocessed forms).

The proteomics dataset was collected from the purified extracellular protein fraction of the biofilm attached to the electrode, and not from the culture medium, meaning that most proteins that are not attached to the biofilm matrix would not be detected. It

can indeed not be excluded that unprocessed forms of the proteins were also detected in the proteomics dataset, with the constraint that they were first purified via the SDS-PAGE 70 kDa band.

3) The physiological interpretation of the OmcZ homolog, based primarily on bioinformatic and structural comparisons, would benefit from a more cautious presentation unless supported by additional experimental evidence. In particular, additional discussion of the proposed mechanism of OmcZ maturation and export in ANME archaea would strengthen the manuscript. Current models for OmcZ-like nanowire assembly in bacteria suggest that the protein undergoes maturation and cleavage prior to polymerization and export, potentially involving type IV pilus/secretion-associated systems. In this context, it would be valuable for the authors to discuss how analogous processes might occur in ANME cells, and how the proposed nanowire structures could facilitate electron transfer from intracellular methane oxidation pathways to extracellular electron acceptors such as electrodes.

We thank the referee for this valuable suggestion. We have already reported on the proteolytic cleavage of OmcZ precursors and the possible responsible serine proteases in lines 173-175 "These proteins were designated OmcZ1Mv (gene ID: DOEMACJB_03675) and OmcZ2Mv (DOEMACJB_03676) and are flanked by two genes encoding serine proteases (Figure 3A), consistent with the presence of OmcZ-specific proteases that are essential for OmcZ cleavage and nanowire maturation". We added a sentence in the same paragraph that Type II/IV secretion systems and Type IV pili may be involved in secretion and anchoring of the nanowire "Further downstream, the genomic neighborhood encodes Type II/IV secretion system proteins and Type IV pili subunits (DOEMACJB_03667-03670) which may be involved in secretion of OmcZ as well as anchoring to the cytoplasmic membrane."

The question how methane oxidation would be coupled to extracellular electron transfer cannot be resolved with the data presented in this manuscript; we would like to abstain from making any ungrounded claims and rather discuss this in a follow-up manuscript where data relating to the molecular mechanism will be collected.

4) I wonder whether relative abundance alone is the best proxy for microbial load or electrochemical activity. In Ouboter et al. (2024), ANME-2d enrichment reached ~82%, whereas methane-dependent current accounted for ~91–93% of the signal. In the present study, ANME-2d abundance increases to 95.8%, but methane-dependent current increases only marginally (to ~97%). Does this simply reflect normal experimental variability, or does

it suggest a more complex relationship between relative abundance and current production?

As discussed in our previous paper, we think that the remaining current that is not methane dependent is likely current produced from *Ca*. Methanoperedens through storage compounds (PHA). Our experimental design does not allow to verify this; in conclusion, we think that 97% is close to the physiological maximum – practically no other processes than methane oxidation lead to current production. We think that the electrochemical data should be interpreted together with the relative abundance data to understand the process of methane-dependent current generation, not either/or.

5) The manuscript reports that Geobacter relative abundance increased from 8.5% in the inoculum to ~18% in the enrichment biofilm after 67 days, before becoming undetectable at the later enrichment stage (119 days) (Lines 116–118). In this context, the explanation that the observed signal originates solely from residual or necromass DNA (Line 120) may not fully account for the earlier enrichment dynamics.

It is noteworthy that the addition of antibiotics directed against *Geobacter* was only started on day 16 (explained in line 525), which explains that *Geobacter* could grow in the first 16 days of incubation, increasing in abundance from 8.5 to 18% and therefore showing higher abundance at day 67 than in the inoculum. As after day 16, antibiotics were regularly supplied, *Geobacter* decreased in subsequent DNA analyses, as is reflected in our data. Therefore, we think that our interpretation of results is sound.

Reviewer #2 (Remarks to the Author):

The manuscript, as it stands now, satisfies all my previous concerns. It is ready for publication.

One minor issue: there is a typo in the Supplementary Figure S6, panel D, the labels of the layer-lines should read l=-2,-1,0,+1,+2 instead of n=...

We thank the referee for their work on our manuscript and appreciate their constructive input. The typo in Suppl Figure S6 has been amended.

Reviewer #3 (Remarks to the Author):

Thank you for the opportunity to review the revision. The manuscript has improved and addresses some of my earlier concerns, including the issue of potential aerobic contamination in the enrichment and the clarification that the filaments were purified from the electrode-associated biofilm rather than the culture medium (as described differently in the previous version).

We thank the referee for their continued work on our manuscript and appreciate their constructive input.

One point I remain uncertain about is the strength of the claims that OmcZ2Mv forms “highly conductive” nanowires. In the section “Structural features optimized for high conductivity,” the authors infer enhanced conductivity/EET primarily from comparative structural features (e.g., inter-heme spacing/packing and surrounding interactions). These are interesting observations; however, it remains unclear whether the proteomics/MS analysis provides peptide-level evidence for covalent c-type heme attachment (e.g., heme-adduct mass shifts on a CXXCH-containing peptide), even though a peptide containing the CXXCH heme-binding motif was detected. The band is heme-positive by staining and the structural work is compelling, but in the absence of clearly documented MS evidence of covalent heme attachment and direct conductivity measurements, some of the “high conductivity/optimized” language appears stronger than what is directly supported.

Due to the positive heme staining of the band and abundant c-type cytochrome binding motifs in the respective proteins we are convinced that the OmcZ protein contains hemes. But it is correct that we did not measure the covalent heme attachment. At this point we don’t know the exact heme chemistry/modifications so

we cannot derive their exact mass so we cannot search our dataset for these peptides. There is also a chance that these peptides never entered the mass spectrometer, because they are too hydrophobic or too large; therefore, biochemical confirmation will need to be performed in future studies.

We changed the wording at several places of the manuscript to reflect our lack of direct data on conductivity.

Relatedly, my question about whether other Ca. Methanoperedens c-type cytochromes were detected in the excised band/fraction is only partially addressed. A clear summary stating whether any additional c-type cytochromes were detected (even at low abundance), or explicitly that none were detected, would be helpful. The response indicates that only an OmcZ homolog was detected, but it remains unclear whether additional (non-OmcZ) c-type cytochromes were present at lower abundance or absent.

We thank the referee for this comment. We have added a sentence to the main text, stating that "Other c-type cytochromes were present at very low abundances (Supplementary Data 2)." The specific data, including rank abundance table and protein identifiers of detected proteins, are provided in the Supplementary Data 2 file.

Modest toning down or qualification of the 'optimized for high conductivity'/'highly conductive' wording would help align the conclusions with the evidence presented. Given the central role proposed for multiheme cytochrome c proteins (including OmcZ homologs) in ANME extracellular electron transfer, I encourage the authors to clarify/temper these statements where needed and ensure that the wording reflects the level of direct support.

We thank the referee for this comment. We have changed the tone where required.
